# From disconnection to compassion: a phenomenological exploration of embodied empathy in a face-to-face interaction

**DOI:** 10.3389/fpsyg.2025.1522701

**Published:** 2025-05-09

**Authors:** Alejandro Troncoso, Antonia Zepeda, Vicente Soto, Ellen Riquelme, Sissi Fuentealba, Catherine Andreu, Ausiàs Cebolla, David Martínez-Pernía

**Affiliations:** ^1^Center for Social and Cognitive Neuroscience (CSCN), School of Psychology, Universidad Adolfo Ibáñez, Santiago, Chile; ^2^Laboratorio de Cognición y Comportamiento Sensoriomotor, Departamento de Kinesiología, Universidad Metropolitana de las Ciencias de la Educación, Santiago, Chile; ^3^Intangible Realities Laboratory, Centro Singular de Investigación en Tecnoloxías Intelixentes, Universidade de Santiago de Compostela, Santiago de Compostela, Spain; ^4^Department of Personality, Evaluation, and Psychological Treatments, University of Valencia, Valencia, Spain; ^5^CIBER of Physiopathology of Obesity and Nutrition (CIBEROBN), Instituto de Salud Carlos III, Madrid, Spain

**Keywords:** compassion, empathic distress, experimental phenomenology, real-life paradigm, empirical 5E approach, micro-phenomenology, embodiment, Alzheimer’s

## Abstract

**Background:**

Research has advanced in revealing psychological and brain mechanisms in empathy-compassion experience. However, much of this research has been constrained by using non-ecologically valid, non-interactive experimental paradigms, as well as a lack of in-depth investigation into participants’ subjective experiences.

**Objective:**

This study aims to bridge these gaps by examining subjective experiences within an interactive setting.

**Methods:**

Forty-two participants engaged in a 90-s, face-to-face interaction with an actor simulating a person with (Alzheimer’s) dementia. The actor’s performance in the interaction followed a validated emotion-inducing narrative about his fear of forgetting their family memories. Subsequently, micro-phenomenological interviews explored participants’ embodied experiences. Data underwent iterative inter-coder processing, and both qualitative and quantitative analyses were conducted. Qualitative analysis focused on temporal dynamics and multilayered dimensions (bodily, affective, attentional, motivational, and relational), while quantitative analysis assessed Bayes’s correlation between phenomenological dimensions and structures of experience, alongside exploratory correlations with empathy-compassion trait questionnaires.

**Results:**

The participants initially connect with the actor’s suffering in their own bodies, leading to an intensified sensation of anguish as the actor vividly describes the fear of forgetting his wife. After, four main experiential ways of navigating the anguish were identified: (1) Relational Disengagement, characterized by detachment from others’ suffering, reduced anguish intensity, and a cold interaffective space; (2) Persistent Angst, characterized by ongoing distress; (3) Anguish Anchoring, characterized by a reactive willingness to alleviate suffering, intense bodily sensations, fluctuating presence, and a less warm, more distant interaffective space; and (4) Compassionate Balanced Support, characterized by a felt presence within a warm interaffective space, motivating balanced support for others. These graded experiences were positively correlated with the ‘empathic concern’ trait assessed by the Interpersonal Reactivity Index scale.

**Conclusion:**

This study suggests a shift in empathy research by proposing moving from a traditional binary view (distress and compassion) to a nuanced framework identifying four distinct and holistic embodied experiences.

## 1 Introduction

Our capacity to connect with and comprehend the experience of others, commonly referred to as empathy, plays a pivotal role in generating prosocial motivation and moral behavior (Decety and Cowell, 2014). Furthermore, connecting with another’s suffering can be both fulfilling, enabling us to offer support and comfort, and demanding, with the potential to lead to persistent personal distress (Klimecki et al., 2014; Thirioux et al., 2016). Empathic experiences that are demanding bear particular significance when they occur regularly, as they can profoundly impact our mental and physical wellbeing, as well as the quality of our relationships (Trzeciak et al., 2017). This understanding is pivotal for grasping the dynamics of caregiving relationships and their professionalization.

The exploration of how individuals perceive and relate to the suffering of others has been a central area of investigation within the fields of social neuroscience and psychology. Two distinct ways of responding to the suffering of others have been identified: empathic distress and compassion (Klimecki et al., 2014; Thirioux et al., 2016). In empathic distress, individuals become more distressed by another person’s suffering than genuinely concerned for the wellbeing of the other individual (Batson, 2011). This heightened distress often blurs the lines between one’s own identity and that of the person in distress (Gelhaus, 2011). In addition, empathic distress is characterized by intense emotional and physical resonance with the other person, a lack of clear distinction between self and other, and a predominantly self-centered perspective (Thirioux et al., 2016). Concerning compassion, it is defined as the emotional response that arises when witnessing another person’s suffering and motivates a subsequent desire to help (Goetz et al., 2010). In addition, compassion is marked by bodily resonance with the other person, a clear distinction between self and other, and a predominantly other-centered perspective (Thirioux et al., 2016). Moreover, the emotive aspect of compassion is directed both inward, allowing individuals to tolerate their distress, and outward, enabling them to respond with genuine concern and a desire to offer help (Singer and Klimecki, 2014). This dual classification has proven indispensable in unraveling the intricate connection between empathic responses and mental health within healthcare and caregiving contexts (Trzeciak et al., 2017).

While empathy research has yielded valuable insights into these types of empathic experiences, its scientific methodology faces two significant limitations that need to be addressed (Gallagher, 2012; Schilbach, 2016): the ecological validity of the non-interactive experimental settings, and the absence of a comprehensive recollection of deep subjective experiences.

Firstly, the methodological approach to empathy research has entailed investigating participants in laboratory settings through passive exposure to predetermined stimuli, such as emotional faces without an active interaction (Fan et al., 2021; Shamay-Tsoory and Mendelsohn, 2019). These studies often examine physiological responses to stimuli, such as reactions to images depicting another person’s pain (Schulte et al., 2022) or to narrative-driven videos (Klimecki et al., 2014).

This traditional laboratory setting narrows the focus of empathy research to isolated brain phenomena, offering precise correlations for methodically dissected elements (Ibanez, 2022). While this experimental setting prioritizes precision and internal validity, it falls short of fully comprehending empathy’s intricacies, relying solely on information processing within controlled, artificial laboratory environments, with limited ecological validity (Fan et al., 2021; Shamay-Tsoory and Mendelsohn, 2019).

In contrast to traditional settings, real-life situations involve complete engagement of the body, encompassing both biological and experiential dimensions, which are often inadequately captured or addressed in controlled experimental paradigms. This engagement manifests through posture, movement, voice, and emotions within a multisensory, dynamic, and authentically natural context (Shamay-Tsoory and Mendelsohn, 2019).

In addition, most of our social experiences involve engaging in mutual interactions. This means that these interactions typically occur within a shared world in which agents are immersed in real-time and the interactions are reciprocal, such that one agent’s expression affects the other and vice versa (Tanaka, 2015; Fuchs, 2017). The absence of this mutual interaction in traditional research is a main limitation because studies on empathy and compassion focus on a unidirectional dynamics perspective, often overlooking the intersubjective dynamics that occur in this context (Redcay and Schilbach, 2019). This is supported by various empirical research findings indicating that interactive settings exhibit distinct neural patterns when compared to passive interactions (For reviews see: Redcay and Schilbach, 2019).

Secondly, in social neuroscience, assessing the experience of perceiving suffering has predominantly relied on state self-report measures, such as questions like “How much empathy did you feel?,” and trait questionnaires, like the Interpersonal Reactivity Index (IRI). It is important to note that these self-reports often serve as the foundation for interpreting research findings (e.g., Klimecki et al., 2014; Timmers et al., 2018). Self-reports are limited in capturing the depth of subjective experiences, making it difficult to fully understand how participants experience the suffering of another. When participants rate the quantity, valence, intensity, or arousal of their empathy-compassion, they appraise their experiences through a rational lens, assigning a numeric value to predefined subjective categories without considering that these elements may fluctuate throughout the experience (Olivares et al., 2015). However, numerous studies have directly addressed the limitations of traditional self-report methods by adopting a phenomenological approach, which has been proposed as a rigorous method for studying lived experience (Berkovich-Ohana et al., 2020). Thus far, several studies have shown that the description of lived experience can be accurate and also very detailed, allowing for a better understanding of empathy and compassion (Coupé and Ollagnier-Beldame, 2022; Przyrembel and Singer, 2018).

The limitations of empathy research described above have recently been addressed by a growing wave of theoretical studies dedicated to integrating the study of empathy into real interactive settings, incorporating the investigation of subjective experiences (Troncoso et al., 2023; Parada, 2018; Shamay-Tsoory and Mendelsohn, 2019; Froese, 2015). For instance, combined proposals rooted in the embodied and enactive tradition suggest the study in natural contexts, where two agents interact dynamically, while simultaneously collecting physiological data and exploring the lived experiences (Troncoso et al., 2023). This methodology is based on the basic knowledge from social neuroscience, phenomenology, and enactivism, that the body (e.g., using free and natural posture in the interaction), context, and active interaction influence the experience of being with another (Fan et al., 2021; Froese, 2015; Redcay and Schilbach, 2019).

Despite these theoretical advancements achieving a more holistic and integrated view of empathy and compassion, to the best of our knowledge, no prior empirical research has incorporated detailed descriptions of empathic experience in a social interaction context.

The current study aims to deepen the knowledge of empathy by exploring how people experience in different ways the suffering of another through experimental psychology in an interactive-ecological context. To do so, we conducted a detailed examination of the lived experiences of participants, focusing on the embodied (rooted in bodily feelings and constraints in the body experience), multi-layered dimensions (including the bodily, attentional, emotional, interpersonal space, motivations, and thought flow, and how these layers interact during the empathic experience) and the temporal dynamics of empathic experiences during an interaction with an actor portraying a person with (Alzheimer’s) dementia. After the social interaction, we employed a second-person methodology (micro-phenomenological interview) to gather experiential data from the participants. Additionally, a secondary objective of our study is to explore the relationship between the phenomenological data and the commonly used state and trait questionnaires for compassion and empathy. This exploratory aim explores the potential of phenomenological analysis in enhancing our understanding of traditional trait questionnaires and self-reports.

## 2 Methodology

### 2.1 Participants

From April 2023 to September 2024, 42 individuals participated in the study, all of whom were Latin American and Spanish-speaking. Inclusion criteria required individuals with no clinical history of cognitive, neurological, or psychiatric disorder and normal or corrected-to-normal visual acuity. All participants gave written informed consent. The study procedure conformed with the Declaration of Helsinki principles and was approved by the “Scientific Ethics Committee of the University Adolfo Ibañez.” The sample size was initially determined based on a previous video-task study with 28 participants that explored empathy for pain settings (Martínez-Pernía et al., 2023). However, we increased it by 50% to 42 participants to account for the study’s complexity and the potential emergence of additional experiential structures. The participants were characterized based on sex, age, and socio-economic status using the AIM questionnaire [Association of Market Researchers and Public Opinion of Chile (AIM), 2023]. AIM uses the Socioeconomic Groups model to measure social stratification, based on three variables: per capita income, formal education, and the occupation of the primary household provider. Additionally, the DASS-21 questionnaire was used to measure depression, anxiety, and stress (Antony et al., 1998), alongside the Interpersonal Reactivity Index (IRI, Davis, 1983), which measures empathy, and the Compassion Scale (CS, Pommier et al., 2020), which assesses compassion.

### 2.2 Construction and validation of the social interaction

In this study, participants interacted with a trainer actor who simulated a person with dementia in a semi-structured interaction. To organize the interaction, we used a structured empathic narrative to guide the performance of the actor. This empathic narrative was validated and utilized by the trained actor in the experimental setting to ensure consistency across participants. To validate the performance, an initial validation was conducted through a video presentation. In this video, the actor responded to questions about his life experiences, specifically focusing on those with a negative valence. The creation of empathy-inducing videos entailed a four-stage procedure: initially, drafting preliminary versions grounded in interviews with three caregivers of Alzheimer’s patients; subsequently, these scenarios underwent evaluation by a clinical psychologist with a specialization in Alzheimer’s disease; the third step involved honing the narratives in partnership with a professional actress; and the final phase comprised the production of four videos, each with a negative emotional valence.

These performances typically spanned around 60 s each. To validate and choose a particular empathic interaction included in the study, a total of forty-one participants (with an average age of 43 ± 15) took part in the validation process. The video evaluation utilized the Self-Assessment Manikin (SAM), to assess emotional valence and arousal (Bradley and Lang, 1994). Additionally, a 9-point rating scale (0 = no anguish; 9 = the most anguish you have ever experienced) was used to gauge the level of anguish experienced. The performance selected for analysis responds to the question, “What do you fear forgetting?.” Within this portrayal, the actor articulates deep-seated apprehensions, principally the fear of losing memories of loved ones, with an emphasis on his spouse, in the context of Alzheimer’s disease. The temporal structure of the performance is deliberately designed to culminate in an emotional apex where the actor reflects upon his spouse, eliciting a representation of emotional anguish. For this study, the chosen narrative yielded a high average emotional arousal of 5.98 ± 2.73, indicating strong emotional engagement. The average emotional valence was 2.05 ± 1.97, signifying a predominantly unpleasant emotional tone, and the anguish rating was notably high at 6.0 ± 2.74 (the narrative is in Supplementary material). The other narratives with different valence levels (neutral, positive, and negative) used in the procedure, but whose data are not reported in this article, followed the same procedure.

### 2.3 Procedure

Initially, participants were asked to complete self-reports (Dass-21, Interpersonal Reactivity Index, Compassion Scale, and socio-demographic variables). Participants were then equipped with a series of physiological instruments (mobile electroencephalography, electrocardiogram, electrodermal activity sensor, and an eye-tracking system). The report of these physiological data falls outside the scope of the study’s objectives. Following this, participants were positioned in a laboratory setting that was adapted with furniture to resemble a living room, awaiting the entrance of the actor. Upon the actor’s arrival, accompanied by a researcher’s assistant who guided him by the arm to the laboratory, the participants were initially instructed to ask predetermined questions that allowed the actor to respond with the validated performance. These questions were presented in six trials, each consisting of two neutral, two positive, and two negative questions, arranged in a pseudo-randomized order. The actor then responded as consistently as possible according to the validated performance described above. To ensure consistency in the actor’s performance, the video was shown to the actor before each question in a separate room, allowing them to recall their previous performance and replicate it during the interaction. Participants were asked not to interrupt while the actor answered their questions. Following the structured interaction, participants were allowed to engage in an unstructured interaction, where they could interact freely (e.g., ask additional questions). This interaction lasted between 1 and 3 min after each trial. This approach was intended to enhance the authenticity of the interaction and to conclude the interaction without leaving a lingering sense of incompletion.

After each trial, participants completed a self-report scale assessing the intensity and valence of the structured interaction (SAM). The specific interaction evaluated in this study was: “What do you fear forgetting?.” This was scheduled at the end of all the interactions to ensure the freshness of memory for the phenomenological interview. Following the interaction based on the question, “What do you fear forgetting?,” lasting approximately 1.5 min, a microphenomenological interview (MPI) was carried out concerning this specific interaction. The complete experimental session lasted no longer than 70 min.

#### 2.3.1 The micro-phenomenological interview

Conducted in Spanish, the MPI was facilitated by a certified interviewer in MPI methods. These interviews were recorded using an audio device for subsequent transcription. To ensure methodological consistency, the interviews adhered to a predefined procedure grounded in MPI guidelines and maintained uniformity during the data collection procedure (Petitmengin, 2006). A vital aspect of the MPI is the continuous shift in the movement of thinking, redirecting it from its habitual content-oriented focus to the how of the unfolding experience. This process requires a temporary suspension of beliefs, judgments, and commentaries about what is being examined (Varela, 1996; Petitmengin, 2006). This process unfolds through various steps within the interview. These include explaining the purpose of the interview, using a format of open-ended questions focused on the *how* and its temporal unfolding, and guiding the participant toward describing the experience itself. Throughout, the interviewer consistently encouraged the evocation of the experience, helping the participant remain connected to it in the moment of description. The utilization of this evocation principle was deemed essential as it aimed to elicit the participants’ pre-reflective descriptions and vividly explore their past experiences, following the principles outlined by Petitmengin (2006).

The general structure of the interview began with an introduction outlining the interview’s objectives and the approach to both asking and responding to questions. Subsequently, the participants were encouraged to evoke their experiences. It was guided by the following prompt:

*“If you agree, you can close your eyes. I invite you to bring to your body and mind the last experience you had with ‘Mr Marcos’ [actor], from when you ask him what he doesn't want to forget, to when he finishes responding. Feel what is happening in each moment. When you are ready, you can tell me.”*

After the evocation, the participant was asked to describe the overall experience in its entirety, from beginning to end, to gain a comprehensive view of the diachronic (temporal) elements of their experience. This was achieved using questions such as: “And after the sensation in your chest appeared, what happened next?” or ‘What happened at that moment?’

Here, we specifically focused on affective and bodily aspects with questions such as: “How did you feel that anguish in your body?” or “That tension—where was it located?” Attention-related aspects were also explored: “Where was your attention at that moment?” or “What were you looking at?” Regarding the flow of thought, we asked: “What were your thoughts like at that moment?” Micro-gestures that emerged were also examined to delve deeper into the experience. For example: “I understood that there was some relief from that anguish. What did you do to relax it?”

Additionally, we included questions about the interaffective space. Since this concept can be difficult to grasp, we guided participants by providing examples, such as the feeling of tension in the atmosphere during a family gathering. We then asked, “At that moment, how did you feel the space between you and Mr. Marcos?” During these zoom-ins on specific moments, the micro-dynamics were also explored through questions addressing their diachronic aspects.

It is important to note that the questions were open-ended and followed the participant’s descriptions. At no point were specific phenomenological elements introduced that were not already mentioned by the participant. This approach was crucial to avoid leading the participant, particularly regarding prosocial motivational aspects. For instance, we did not ask, “Did any motivation arise?” as this could introduce social desirability bias, which is a common concern in such paradigms. After the interview, participants were asked follow-up questions after the interviewer provided a summary of their descriptions. Questions included: “Do you feel that the description of your interview reflects what you experienced during the interaction?” Participants were also invited to share any additional descriptions. Finally, the interview ended with expressions of gratitude and a formal closing. To review an interview example, please refer to Supplementary material S1.

The interviews averaged 20 min and 27 s ± 4:02 min, totaling 14 h and 18 min. A total of 98,577 words were analyzed, with an average of 3,747 ± 742 words per interview. To ensure efficiency, interviews were conducted immediately after the interaction to maintain the freshness of recall, focusing solely on the selected 90-s interaction and following a systematic structure to thoroughly explore all dimensions and phases. The interview protocol was pilot-tested before the study and further refined as data collection progressed.

### 2.4 Phenomenological analysis

**Figure 1:**
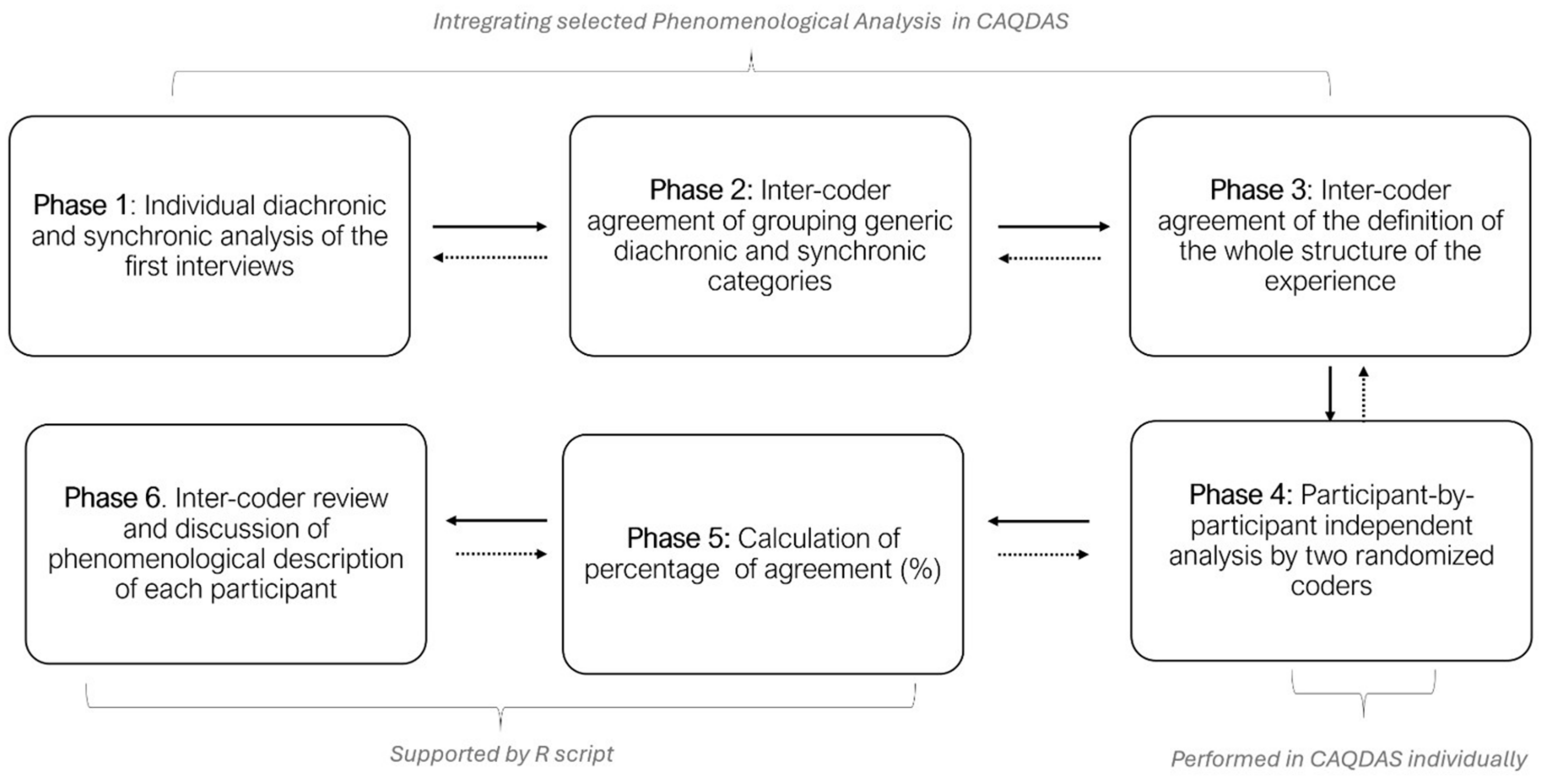
Description of the phenomenological data analysis. The dashed arrows indicate iterative processing.

To conduct the comprehensive phenomenological analysis of our data, we employed an adapted approach based on the MPI analysis method (Valenzuela-Moguillansky and Vásquez-Rosati, 2019) that has been employed in previous research (Martínez-Pernía et al., 2023; Troncoso et al., 2024). This methodology primarily focuses on the comprehensive analysis of the global diachronic and synchronic aspects of the experience. It aims to discern distinct patterns among individuals, integrating both diachronic and synchronic aspects shared among participants, referred to here as distinct experiential structures. To ensure the robustness and consistency of our phenomenological investigation, we employed a novel iterative method within the triangulation process, complemented by an inter-rater agreement index (Troncoso et al., 2024). In this analysis, we utilized Computer-Assisted Qualitative Data Analysis Software (CAQDAS) to facilitate the organization of descriptive statements and the subsequent analysis. Each researcher conducted independently an analysis following a consensual work structure, which included the temporal phases of the experience (diachronic dynamics), the phenomenological categories, and the experiential structures. Two coders from a randomly assigned group of three coders analyzed each interview separately. By exporting the results from CAQDAS into an HTML report generated via the R environment, we identified disagreements among all subjects in each phase and each specific category. In an iterative process, the researchers reached a consensus for each subject, striving to achieve the highest level of agreement possible. It is important to note that all categories were emergent and triangulated, rather than being pre-established. We have summarized the most important steps of our phenomenological analysis in Figure 1. The average percentage of agreement was 81%. Additionally, the agreement was complete in the experiential structures. This value indicates a high level of consensus among coders. For a more detailed description of these procedures, please refer to Supplementary material S1.

For a more detailed review of the triangulation procedure and results by subject, please refer to Supplementary material S3.

### 2.5 Quantitative analysis

To gain a more comprehensive understanding of the phenomenological experience, we conducted a quantitative analysis of phenomenological attributes. Furthermore, we explored the association of the main phenomenological categories and the experiential structures with questionnaires based on empathy-compassion traits. Below, we show the questionnaires and the quantitative transformation of the phenomenological data. For empathy-compassion traits, the interpersonal reactivity index (IRI, Davis, 1983) and compassion scale (CS, Pommier et al., 2020), were used. The IRI assesses empathy through four subscales: Perspective-Taking, Fantasy, Empathic Concern, and Personal Distress. The CS measures compassion across four dimensions: Kindness, Common Humanity, Indifference, and Mindfulness (Pommier et al., 2020).

For quantitative analysis of phenomenological data, a proportion of participants associated with each main experiential dimension and its corresponding sub-categories were calculated. Only categories that exhibited full agreement among the researchers were included in this analysis.

Additionally, given the graded nature of both the sub-categories and the structures of experience that emerged in this study, they were both transformed into an ordinal scale. This procedure is similar to that carried out in other studies (Nave et al., 2021). Here, for each participant in the specific phase analyzed, the categories were automatically transformed into numbers. For example, for a category called “intensity,” when participants experienced low intensity, it was assigned a value of 1. When they experienced medium intensity, it was assigned a value of 2, and when they experienced high intensity, it was assigned a value of 3. Since these categories were identified for each participant and their corresponding phase, the transformation was carried out automatically.

A Bayesian correlation analysis was conducted to investigate the relationship between phenomenological and self-report data. This method was chosen for its robustness, especially in the context of research with a small sample size (Wagenmakers et al., 2018). Bayesian correlation analysis offers advantages over classical correlation analysis by allowing the incorporation of prior knowledge, which is particularly valuable when there is empirical information about the expected direction of the correlation (Wagenmakers et al., 2018). Firstly, to assess the degree of correlation between the main phenomenological categories and the structure of experience, Bayesian Kendall’s Tau correlations were performed. Secondly, as a secondary aim of this study, Bayesian Kendall’s Tau was included to analyze the correlation between the main phenomenological categories and the structure of experience with questionnaires (CS and IRI).

Quantitative analyses were conducted using the R statistical programming environment and Bayesian Correlation was conducted using JASP (Version 0.18.1).

All quantitative results are openly accessible in HTML format in Supplementary material S4.

## 3 Results

### 3.1 Sociodemographic characteristics and empathy/compassion traits

A total of 42 individuals participated in the study. The socio-demographic description and the results of CS and IRI are provided in Table 1.

**Table 1:** Sociodemographic and empathy/compassion questionnaires results of participants.

Variable	Sample, *N* = 42^1^
Age	24.0 ± 7.8 years
Sex	
Male	30.95%
Female	69.05%
Socio-economic status	
Low	13.50%
Middle	75.60%
High	10.80%
Dass-21	
Depression	6.9 ± 9.4
Anxiety	9.6 ± 10.1
Stress	12.6 ± 10.4
Compassion scale total	68.4 ± 5.4
Kindness	8.5 ± 6
Common humanity	9.1 ± 6.7
Indifference	4.0 ± 3.5
Mindfulness	9.0 ± 6.3
Interpersonal reactivity index total	73.4 ± 13.2
Fantasy	19.2 ± 5.0
Perspective taking	20.2 ± 4.6
Empathic concern	22.3 ± 5.0
Personal distress	11.4 ± 5.3

1 Mean ± SD; Percentage (%).

### 3.2 Phenomenological results

This section presents the results, organized into phenomenological categories, diachronic structure, and four experiential structures, which will be explored in the following subsections: 2.1 Phenomenological Categories, describing the experience through categories and subcategories that emerged during the analysis, with four overarching experiential categories identified: bodily resonance, interaffective space, interpersonal presence, and dis/engagement acts; 2.2 Diachronic Structure, describes the temporal unfolding of the experience across three distinct phases: connection with the suffering of the actor, sensing the climax of anguish, and navigating the anguish; and 2.3 Four Experiential Structures, which represent the ways participants navigated the anguish, revealing four distinct structures: relational disengagement, persistent angst, anguish anchoring with other-oriented support for suffering, and compassionate support for suffering.

#### 3.2.1 Phenomenological categories

**Figure 2:**
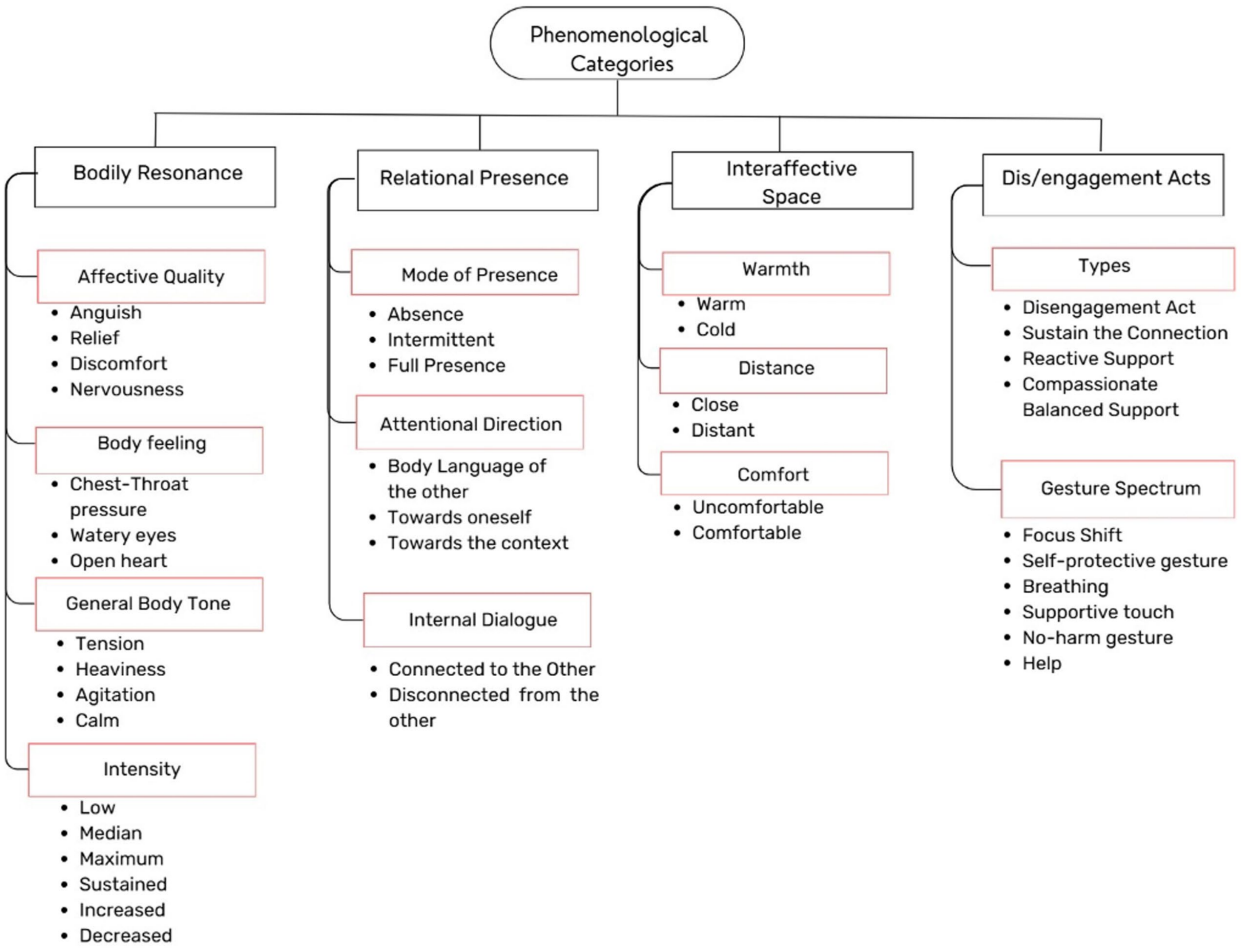
A schematic representation of the experiential categories identified is presented. The main categories are highlighted in black, with sub-categories indicated in red.

This section outlines the four main categories identified in participants’ experiences, along with the subcategories that comprise them (Figure 2). For a detailed description, including its importance, reflection, and supporting textual quotations, please refer to Supplementary Codebook. Additionally, since the descriptions and the number of participants for each category vary across the temporal phases analyzed, we provide only a general overview here. For detailed information on the number of participants associated with each subcategory in specific phases, please refer to Section Dynamic Lines in the Supplementary material S4. The distinct ways participants experienced these phenomenological dimensions are further elaborated in the results section on the Four Experiential Structures.

##### 3.2.1.1 Bodily resonance

At the beginning of the interaction and during its most intense moments, participants felt deeply connected to the actor, experiencing their suffering within their bodies. This connection was in sync with the actor’s distressing performance. The affection expressed by the actor resonates in the participants’ bodily sensations, leading to a diverse spectrum of embodied emotional responses closely synchronized with the actor’s performances. These body-affective experiences were dynamic and changed as the performance evolves, as well as when the internal experience changed. A common element among participants and across the different moments of the experience was that most reported feeling localized pressure in their chest and throat regions. The intensity of this sensation varies from subtle to intense, closely mirroring the affective quality of the actor’s anguish. Additionally, for some participants, this sensation was accompanied by a feeling of tearfulness during the most intense moments.

[Referring to the moment when the actor says he does not want to forget his wife] *“What I was telling you, like from here in the chest, well … I don’t know how to explain it properly, but it was like something that wanted to rise … Strong, quite strong. And, well, it was like a desire to cry—I’m very emotional—but it felt like holding back tears, like everything was tense, and the sensation in my chest was as if it wanted to come out.”* (S19)^1^

Alongside this bodily sensation, an overarching sense of bodily tension pervaded the participants’ experience. In addition, the predominant affective quality during the initial phases of the interaction was anguish, described as a distressing sensation.

[At the beginning of interaction] *“The anguish…in the whole body. Yes, in the whole body, because at that moment I remember that I felt, I mean I felt like vibrations in my body”* (S2)^2^.

Various other affective qualities were identified at different stages and in distinct ways of experiencing (details on these differences will be provided later). These include feelings of discomfort and nervousness, as well as a sense of relief accompanied by a bodily tone of calm. The latter was observed in some participants during the final phase of the interaction.

[Referring to the final phase of the interaction] *“It’s like everything I told you kind of settles as it all assimilates, and the tension I just felt slowly, slowly starts to fade. That cold sensation I felt also seemed to go down like the feeling diminished. It starts to disappear … I think I straightened up a bit to thank Marcos … I felt like it was a closure.”* (S8)

In general, the intensity of this resonance varied from low to high, depending on the temporal evolution of the interaction and each subject’s way of experiencing the actor’s suffering. It was also described in terms of its dynamic, with participants noting an increasing intensity at the beginning, which grew as the interaction progressed.

[Referring to the shift in resonance intensity] *“That was like the peak like it kept building up, and then when he said it was someone really important and that he didn’t want to forget her … I felt like I got more nervous. That was the most intense moment of the conversation. I felt like I was sweating a bit more, feeling more nervous, like I didn’t know how to respond to him … I felt like I got more tense.”* (S11)

Additionally, some participants described these bodily sensations as persisting in intensity, while others mentioned that they diminished in the final phase of the interaction.

[Referring to the shift in resonance intensity] *“I think it was, like, it was clear. At first, it was very intense—when he said it, it really hit me—but then, as I started thinking about other things, it began to diminish a little.”* (S4)

##### 3.2.1.2 Interaffective space

The participants experienced the emergent space within the actor-participant dynamic as a variable affective ambiance. They reported feelings ranging from a comforting sense of warmth and closeness to discomfort and distance. Regarding the comforting and warm aspects of the interaffective space, participants described it with a sense of welcoming familiarity.

*“I didn't feel that the ambiance was tense. I feel like it was pretty fluid within how controlled the space was. Eh. But I feel it was very fluid. As I said, I think I, I felt like I could have even sat down and talked with him for a while… I felt very close… the space actually, this space is comfortable for me”* (S17).

Conversely, some participants felt the ambiance was cold, uncomfortable, and distant. Their descriptions featured a spatial depiction where, despite physical proximity, the interaffective space was emotionally distant and disconnected.

*“I felt it like a little bit more like the ambiance between the two of them was very tense. And well, like distant”* (S19).

##### 3.2.1.3 Interpersonal presence

In the interaction, participants experienced varying qualities of presence, which can be described as the degree to which they felt fully engaged and connected to the moment of interaction. This spectrum of presence spans from moments of feeling somewhat detached or absent to instances of being completely immersed and attentive, with intermittent periods of engagement. These distinct qualities of presence are grounded in three fundamental subcategories: the focus of their attention, the internal dialogue happening within their minds, and imagination. (To understand the importance of this category in the phenomenon under study, visit the codebook in its section “Main category: Interpersonal Presence, Importance”).

The sensation of absence arose as a feeling of being disconnected from the interaction, one’s attention drawn elsewhere, beyond the realm of the actor. Moreover, internal dialogues tended to detach from the present moment, often meandering toward thoughts about personal family matters or the context of the actor.

*“Eh like this voice-over, but my presence was set in like in a kind of review of my life…My body my attention in another space mm. Another space. No, no, here, less…Less attentive to this place and less attentive to the dialogue”* (S26).

Intermittent interpersonal presence manifested as a continuous oscillation between being absent and being fully engaged. Participants alternately immersed themselves in the actor’s experience and then redirected their focus toward other elements.

*“For a few seconds I remembered my girlfriend, and then my mind kind of went a little bit to have her present. And then I quickly went back to what he was telling me”* (S12)

In the domain of full presence, participants found themselves immersed in the narrative of the actor, closely attuned to their bodily expressions. Active listening became a hallmark, characterized by minimal internal dialogue interruption.

*“All of it. I mean, sometimes I get the sticks because I'm average. But my whole 100% attention was on him”* (S9).

##### 3.2.1.4 Dis/engagement acts

The dis/engagement act manifested as a vivid, pre-reflexive gesture aimed at nurturing or weakening the interactive exchange with the other person. These gestures served as active strategies not only to strengthen or diminish the interaction but also to address and manage the suffering of both the actor and the recipient. Those acts of dis/engagement varied among participants and were categorized into disengagement, maintenance of mental stability, reactive support, and compassionate balanced support.

Some participants engaged in conscious acts involving a deliberate redirection of their attention away from the interaction. This phenomenon is characterized as an act of disengagement that emerged as a way to cope with a high intensity of anguish. It was not a form of disrespectful disconnection or indifference toward the actor.

*“When I am in that moment as if I am disconnected, I try to just comment, to abstract myself from everything I am feeling at that moment”* (S21)

Conversely, participants managed to uphold their inner equilibrium and maintain their interaction with others despite the distress they might have been feeling. This act involved employing intentional strategies such as focused breathing, attending to sensory perceptions in their hand, suppressing the crying and deliberately controlling emotional responses.

*“I felt like crying, but I was still concentrating on what I was saying. And that was like a strategy not to cry. One of the strategies I used to cry…I kind of gasped twice and it went away, I did all that at once”* (S4)

Another act of vinculation is reactive support. It is described as a sense of motivation to provide comfort to the actor, either through physical touch or supportive words, all nurtured by a sense of distress. This form of support is associated with an immediate response to the other person’s urgency or desperation, either through physical touch or supportive words. It is characterized by feelings of a sense of urgency, leading to the willingness to act such as touching or approaching quickly to alleviate perceived suffering. Unlike other acts of vinculation, its voluntariness is unclear, as it appears to be more passive and seemingly involuntary. It is driven primarily by the perception of distress, rather than by a conscious effort to regulate one’s emotional response and provide support.

*“I felt sorry for him. I felt like hugging him, but obviously, I couldn't. I saw him as if he were my dad, so I empathized with the situation. I saw him as if he were my dad, so I empathized with the situation and I was kind of sad”* (S7)

Conversely, some participants perceived their engagement as acts of compassionate support while maintaining inner equilibrium. In these instances, their motivation was focused on offering comfort, whether through physical touch, choosing not to intervene further, or simply being present to ease the actor’s suffering. Some participants actively consoled, while others listened respectfully, aiming to alleviate pain through their presence alone. In both cases, their actions were grounded in emotional stability and self-control, with a clear and deliberate intention centered on the wellbeing of others while managing their own emotions. This act could also involve the use of regulation strategies, allowing individuals to maintain emotional balance and effectively support others while addressing their own emotional needs.

*“Despite feeling a bit of distress from experiencing this compassion so intensely, I also feel comfortable with this emotion, but at its core, it’s like a kind of tenderness… and in those moments, it’s about offering some support, really validating what he’s feeling, and it doesn’t become uncomfortable for me”* (S18)

#### 3.2.2 Diachronic dynamics

**Figure 3:**
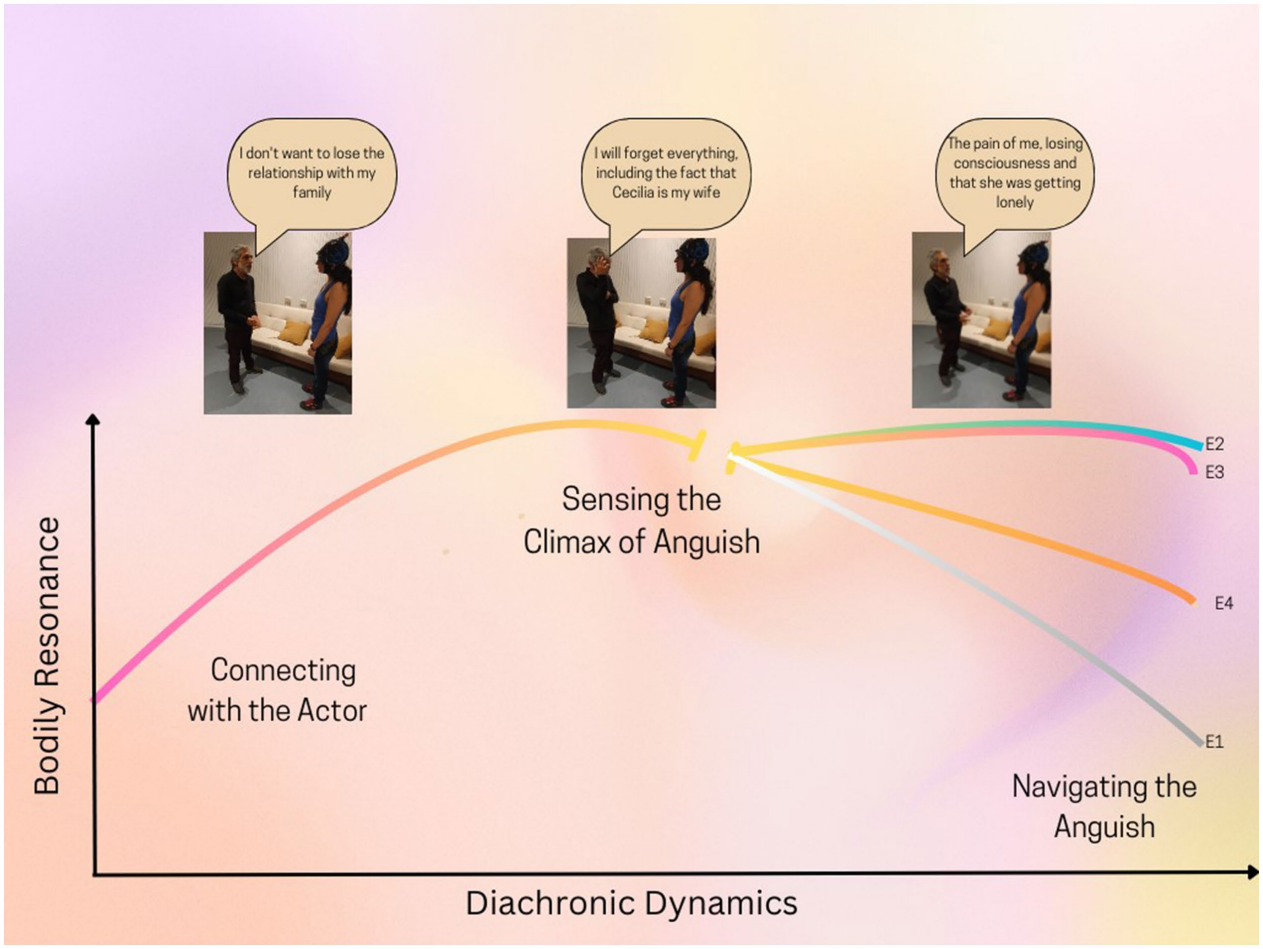
The diachronic dynamics illustrate how the intensity of bodily resonance evolves through three phases, closely synchronized with the actor’s performance. Following the climax of anguish, four distinct experiential structures (Experience 1 to Experience 4) emerged, each representing a specific quality of bodily resonance in navigating this anguish. “E” denotes the experience, with its corresponding number indicating the type of experience.

The diachronic dynamics represent the temporal dimension of the experiences, illustrating how the experiences change over time (Figure 3). Three phases were described by the participants in this study. Initially, in the phase called “Connection with the suffering of the actor,” the participants connected to and felt the suffering of the actor in their bodies. Participants described a sense of anguish (73.8%), discomfort (11.9%), and nervousness (14%) during this moment, each of which was felt as a distinct quality in their bodily experience. The anguish was particularly pervasive, often manifesting as a tightness in the chest and throat.

The affective-bodily feeling was experienced in certain participants with moderate intensity (38%) and, in certain instances, gradually intensified over time, particularly as the actor’s narrative became sharper (47%), which was felt as an increase in the signs of suffering in their response.

[At the start of the narratives] *“I start to feel like the emotions, I start to feel sad because I was reminding him that he is going to forget. I started to feel my body more tense. More rigid”* (S4).

In addition, the participants focused mostly on the actor’s body language (35%), and some focused on one aspect of the inner experience more than the other (23%).

The subsequent phase, known as the “Sensing the climax of anguish” involved a swift escalation of the anguish sensation when the actor vividly described and connected with the fear of forgetting his wife. The entire sample (*N* = 42) described the same pivotal moment, marked by the peak of intensity in the performance when the actor uttered, “I will forget everything, including the fact that Cecilia is my wife.” This moment marked the shift from a moderate to a high level of intensity (92%), with a notable increase in participants reporting feelings of anguish (95%), often experienced in the chest and throat area (88%). Some participants described a sensation of teary eyes (23%), while others reported a feeling of sadness in their faces (9.5%).

*[Talking about the Wife] “When he started talking about his wife, of course, it was kind of sad, but when he said he was going to leave her alone, it was like a lot of pain in my chest, I felt more tense and everything”* (S4).

This moment was also characterized, for the majority, by a strong sense of presence in the interaction (52.8%), while others experienced fluctuations, alternating between being fully present with the other and moments of absence (33.3%).

Following, the phase known as “navigating the anguish” participants reported a range of experiences, from feelings of disconnection (14.3%) and persistent anguish (31%) to the emergence of the intention to offer support in a reactive manner (21.4%) and in a more balanced way (33.3%). The intricacies of those four experiential structures will be detailed in the following section, underscoring the structures of experiences found. In this phase, we uncovered the primary distinctions that set it apart.

#### 3.2.3 Four experiential structures

**Figure 4:**
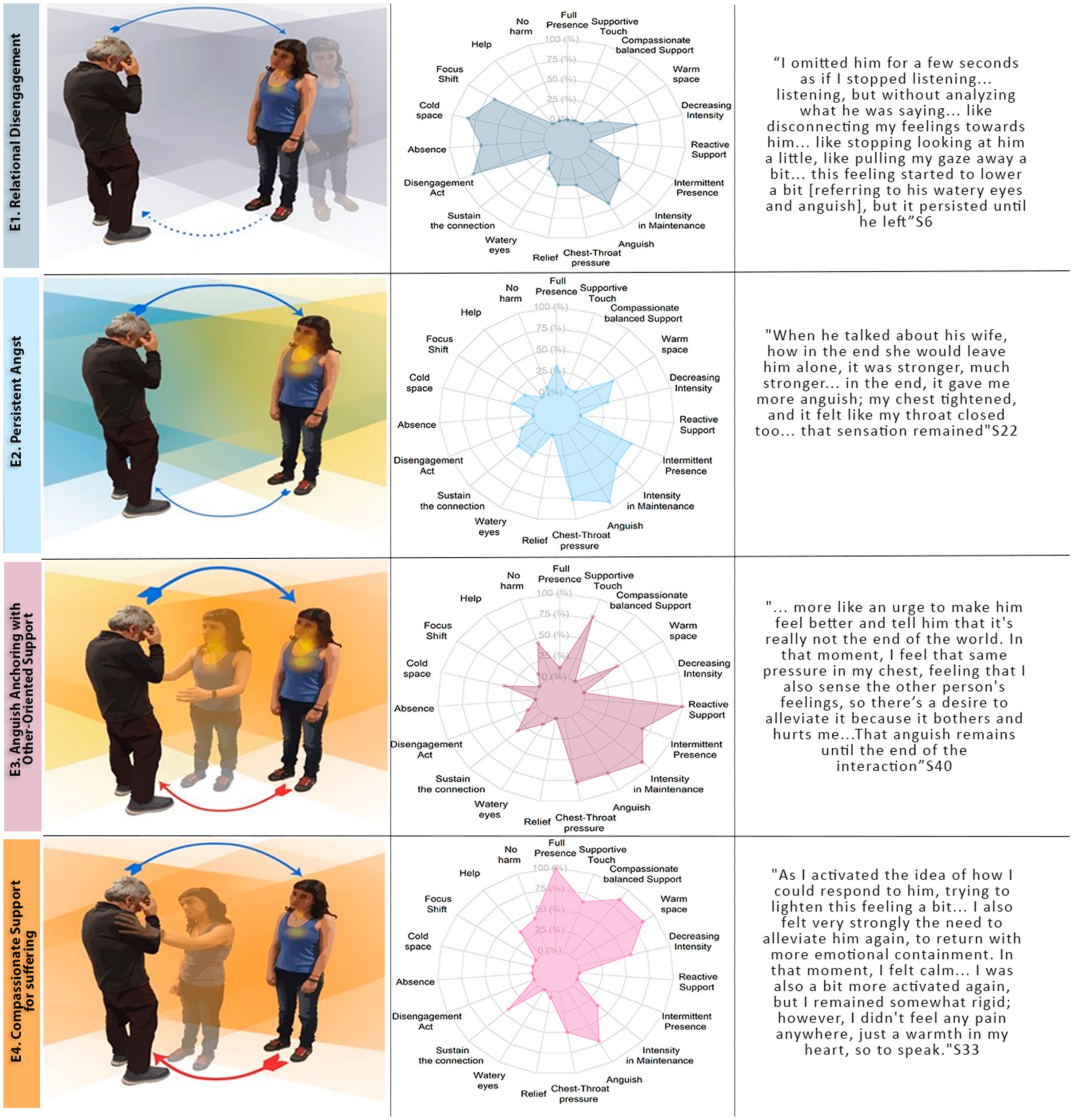
A comparative analysis of the four experiential structures. The left panel provides a qualitative depiction of the distinct experiences, highlighting the key differences between them. The middle panel shows the percentage of participants within each experiential structure who reported experiencing the corresponding phenomenological dimension. Additionally, the categories selected in these graphs were those that best distinguish the experiential structures. For more details and examples of each category, please refer to the ‘Phenomenological Dimension’ section. The right panel complements this by showcasing exemplary descriptive statements. S = Subject.

The four experiential structures were identified based on how participants experienced bodily resonance, interaffective space, interpersonal presence, and engagement acts, specifically while navigating the anguish phase. The resulting four experiential structures encompass relational disengagement (Experience 1, E1), persistent angst (Experience 2, E2), anguish anchoring with other-oriented support for suffering (Experience 3, E3), and compassionate support for suffering (Experience 4, E4). For a qualitative and quantitative summary, refer to Figure 4.

##### 3.2.3.1 E1. Relational disengagement (*n* = 6)

*“I was looking at the floor, just looking at the floor, and then I stopped looking at it. I cut that connection like that. Not disrespectfully, but, well, not empathetically, so to speak.”* (S22).

Six participants followed this experiential structure, representing 14.3% of the sample. Initially, participants shared a feeling of connection with the actor, embodying this anguish in the chest-throw area. During this phase, they engaged deeply with the actor, experiencing a sense of vivid, full presence. Subsequently, participants encountered the peak of anguish resonance, characterized by intense feelings in the chest-neck and eye areas, particularly when the actor expressed fear of forgetting their spouse. Participants then reported a shift, describing a detachment from the actor’s interaction. This active gesture of detachment represented the specific transitional event that initiated Phase 3. In this phase, the detachment was accompanied by a reduction in the intensity of angst (*N* = 3, 50% of the E1) or a sense of relief (33%), often achieved through a purposeful shift of focus (66%) (e.g., looking away from the actor). This detachment was not merely a passive reaction but can also involve deliberate strategies, such as redirecting attention or mentally reframing the situation, to regulate emotional intensity. The quality of presence was mostly perceived as absent (66%) or with intermittent fluctuations between being present and absent (33%). This change in presence was accompanied by a focus primarily on one’s own experience, but also, in some cases, on the general context of the actor (16%). Additionally, some participants experienced fluctuating attention between these two foci (33%).

Furthermore, there was a significant internal dialogue directed toward disconnected elements of the actor’s experience (66%). In addition, the interactive space was perceived as distant (83%) and, for some, uncomfortable (16%).

Given that this structure is the one that most differs from the others, its unique characteristics of absence, the gesture of detachment, and the reduction of bodily sensations were the key features that allowed the identification of participants within this structure.

##### 3.2.3.2 E2. Persistent angst (*n* = 13)

*[Talking about the moment when the actor describes the fear of forgetting his wife] “When the woman showed up out of the blue, it's like my whole body tensed up. The tightness was most pronounced in my chest, and my jaw, I had it clenched… It persisted until he left”* (S13).

This experiential structure involved 13 participants, accounting for 31% of the sample. In Phases 1 and 2, participants’ experiences closely aligned with those described in E1. Following this, they continued to sustain intense anguish, articulating a vivid sense of being affected by the actors suffering in Phase 3. This affective quality was accompanied by an environment that was either cold, distant, and uncomfortable (*N* = 3, 25% of the E2) or warm, close, and comfortable (50%). Participants described an intermittent presence (63%), characterized by internal narratives focused on the actor’s experience (53%) but also their own experience (30%).

Some participants experienced a state of full presence, marked by active engagement and attentiveness to the actor’s body language as the actor described their experiences. Although the persistence of emotions was common, some participants showed a slight decrease in their sensations, accompanied by deliberate regulatory strategies that helped reduce this intensity, such as refocusing on the actor (23%) and deep breathing (15%).

The key characteristic of this structure was the absence of any clear transitional point between Phase 2 and Phase 3, as shown by the lack of an internal gesture that would shift the experience either toward disconnection or an intention to alleviate. This, along with the persistent anguish, allowed it to be differentiated from the other structures.

##### 3.2.3.3 E3. Anguish anchoring with other-oriented support for suffering (*n* = 9)

*“Even his voice quivered a bit, and his eyes welled up [Talking about the actor], and I wanted to reach out and, even though I didn't know how to comfort him and tell him that everything would be okay, maybe, even if it might not turn out that way. But comforting him, that I could do …… It felt like this overwhelming tension, the tension was building up right here (pointing to the chest)”* (S11).

Nine participants followed this experiential structure, accounting for 21.4% of the sample. After establishing a connection with the suffering of the actor in Phase 1 and experiencing intense anguish in Phase 2, similar to E1 and E2, participants continued to endure this overwhelming sensation of anguish (*N* = 9, 100% of the E3).

Beneath this distress, the intention to provide urgent support emerged. This emergence of intention served as the key transitional event that globally marked the beginning of Phase 3. In this phase, this act of reactive support was not accompanied by strategies to reduce distress or maintain the full focus on the interaction. The anguish in this phase was felt, primarily in the chest and throat (78%), maintaining a high level of intensity (67%). This affective quality was accompanied by two distinct perceptions of the interaffective space. Some participants described the interaction as warm (15%), comfortable (22%), and close (22%), while others perceived the interpersonal space as distant (33%) and uncomfortable (22%).

Engagement manifested primarily through the motivation for physical touch, including embraces and gentle caresses (78%), as well as a commitment to causing no harm (44.4%) (e.g., asking more questions). As well as a commitment to causing no harm (44.4%) (e.g., asking more questions). This category reflects the intention to avoid inflicting further harm, often described as the deliberate choice to refrain from probing deeper to prevent exacerbating the actor’s suffering.

These actions were characterized by a sense of urgency to alleviate the suffering of another (100%). Most participant’s experience was marked by an intermittent presence (67%), shifting between images or thoughts about their own experience and the surrounding context.

Three key features distinguish this structure from the others. First, the sustained high intensity of bodily resonance sets it apart from Structures E1 and E4. While it shares similarities with Structure E2, the emergence of a supportive gesture differentiates it. Additionally, the intermittent nature of presence distinguishes it from the subsequent structure, as does the specific engagement act. Together, these elements form the defining characteristics that separate this structure from the one that follows (E4).

##### 3.2.3.4 E4. Compassionate support for suffering (*n* = 14)

*“Smile at him. When I smiled at him, he would smile back at me… It was as if I were this big, protective presence, and he was like a baby, and I was his source of comfort”* (S17).

This experiential structure involved 14 participants, accounting for 33.3% of the sample. After forming a connection with the suffering of the actor in Phase 1 and experiencing profound anguish in Phase 2, participants described an intention to offer compassionate support to the actor. This intention was a defining global element of the transitional event that marked the entry point into Phase 3. Acts of compassionate support are primarily expressed through physical touch—hugs and comforting gestures (*N* = 9, 64% of the E4). Additionally, participants described a “no harm” motivation, evident in 42% of cases, where they chose to remain composed or refrained from asking further questions to avoid intensifying the actor’s suffering. Unlike previous experiential structures, participants in this group successfully maintained their emotional balance while engaging in compassionate acts (100%).

These actions were focused on the actor’s needs, but the intention was more measured and gentle. The response was not driven by an intense emotional reaction but by emotional regulation, allowing for closeness and support without the urgency to act. The intention was clear and deliberate, prioritizing the other person’s wellbeing while managing their own emotions in the process. This quality of this intention marked a key distinction, setting it apart from E3. This was reflected in the quality of bodily resonance, the affective space, and the participants’ presence. The resonance of anguish diminished for the majority of participants (57%), while for others, it remained at a moderate intensity. This affective quality was paralleled by an emotional perception of the ambiance, with 85% of participants describing the interaction space as warm, comforting, and close.

A key distinction of this structure compared to the E3 was the marked sense of full presence (100%). This engagement was demonstrated by participants’ keen attention to every nuance of the interaction, especially the subtle changes in the actor’s body language (68%). Their accounts captured a rich array of the actor’s bodily expressions and the emotions these expressions convey.

#### 3.2.4 Correlation between phenomenological categories

**Figure 5:**
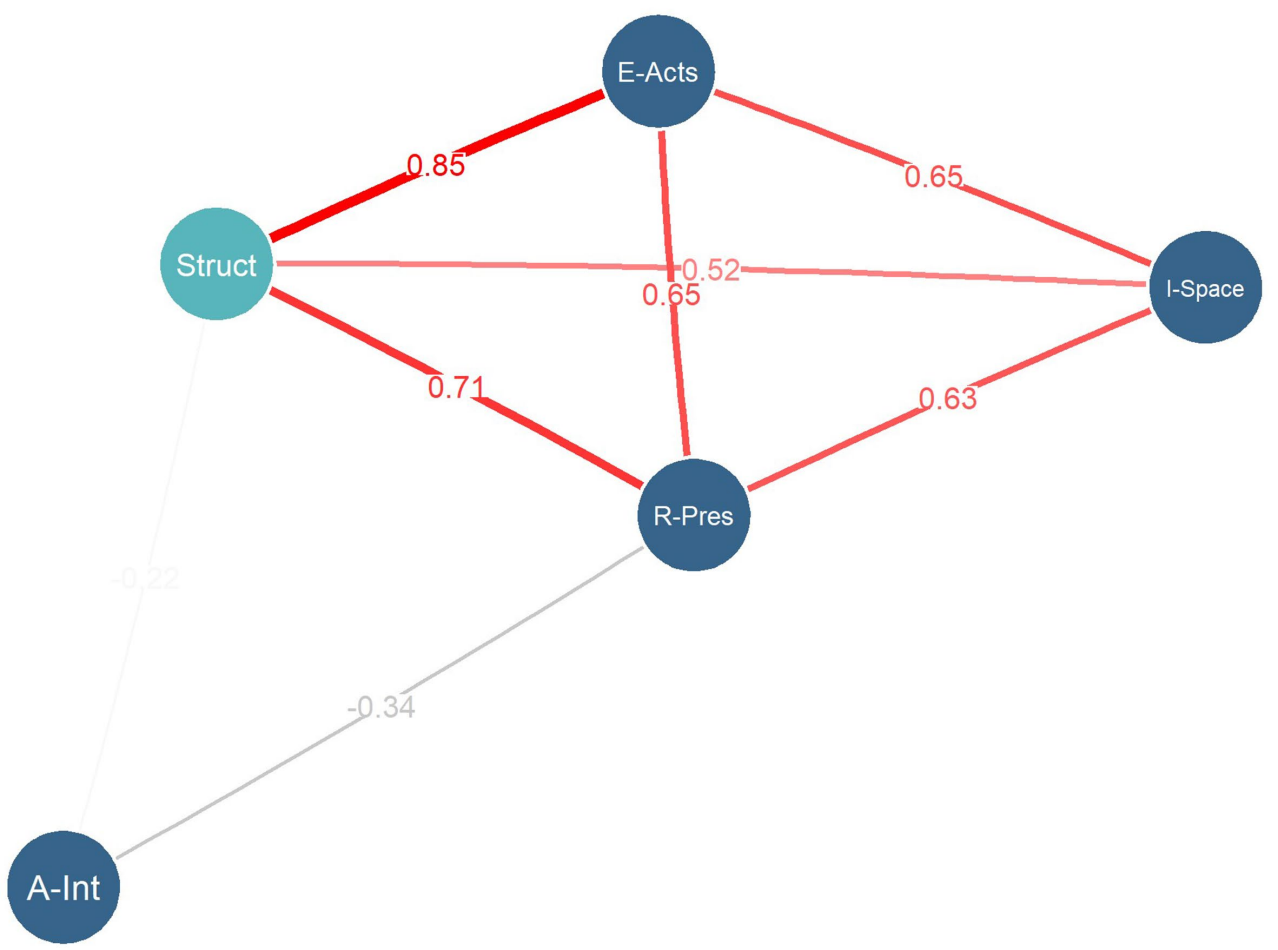
Network graph illustrating the supported Bayesian Kendall’s Tau correlations between phenomenological categories and the structures of experience (Bayes Factor > 100). The distances between nodes, along with the width and color of the edges, indicate the strength of the correlations. Red edges denote supported positive correlations. Black edges denote supported negative correlations. The magnitude of the Bayesian Kendal’s coefficients is displayed at the midpoint of the edges. R-Pres = Relational presence, E-Acts = Engagement Acts; A-Int = Affective Intensity, I-Space = Interaffective space; Struct = Structure of Experience.

We identified a gradation within four categories, which were transformed into numerical values. These categories included intensity of bodily resonance, ranging from low to high (low: 1, medium: 2, high: 3); relational presence, ranging from absent to fully present (absence: 1, intermittent: 2, full presence: 3); interaffective space, ranging from cold to warm (cold: 1, warm: 2); and engagement acts, ranging from disconnected to compassionate (detachment: 1, maintenance: 2, reactive support: 3, compassionate: 4). Similarly, the experiential structures were transformed into numerical values, ranging from the most disconnected to the most compassionate (E1: 1, E2: 2, E3: 3, E4: 4). We calculated the Bayesian Kendall’s Tau correlations between the phenomenological categories (presence, interaffective space, bodily resonances in terms of affective intensity, and acts of engagement) in the navigating the anguish phase, which revealed predominantly strong and positive correlations (Figure 5). The correlations observed were both within the main categories and between the main categories and the structure of experience, except for the subcategory affective intensity of bodily resonance. The correlations between categories ranged from Kendall’s tau = 0.52 (interaffective space—structure) to tau = 0.85 (engagement acts—structure), indicating strong associations across the dataset. The correlations between categories and the structure of experience ranged from tau = 0.52 (interaffective space) to tau = 0.85 (engagement acts).

In summary, a more compassionate structure was linked to a fuller presence, a warmer and more interactive space, and more compassionate engagement acts. However, the correlation with affective intensity was weaker, suggesting a distinct pattern for this subcategory.

#### 3.2.5 Exploratory analysis: correlation between phenomenological data, empathy-compassion trait, and self-report

Subsequently, we assessed Kendall’s Tau correlations between categories and the structures of experience with the trait questionnaires and state self-report. We found a moderate positive correlation between the structure of experience and the IRI empathic concern subscale (which measures the tendency to feel concerned for others in distress) (rs = 0.38; BF > 100), indicating strong evidence in favor of the association. This subscale measures the tendency to feel concerned for others in distress (e.g., “*I often have tender, concerned feelings for people less fortunate than me*”). In contrast, a weak correlation was observed with the IRI total score (rs = 0.293; BF > 10), suggesting moderate support for this relationship. Non-supported correlations were found between the categories and the structure with CS (e.g.*, “When I see someone suffering, I feel a strong desire to help them and make them feel better.”*). For detailed results, see Supplementary Table S1.

## 4 Discussion

The study aims to enhance our understanding of empathy by investigating diverse ways individuals experience the suffering of others. This was achieved through a research design that integrates the phenomenological method within experimental psychology, specifically within an interactive ecological context. The primary outcome and significant contribution of this study is the delineation of a graded empathy spectrum which ranges from relational disengagement and persistent angst to anguish anchoring with support, and finally, compassionate support for suffering.

This spectrum distinctively captures how vividly participants encompass the embodied and interactive dimensions of being in tune with the suffering of another. The embodied and interactive dimensions are distinctly evident in the interaction process. Particularly in terms of how they vividly experience the motivation to either support or detach and how they sustain presence or engagement with the actor. In addition, the structures also show differences in affective quality in how they feel the suffering of another within their bodies and how they feel about the ambiance or space of the interaction.

In this discussion, we address three main points: the differentiation of experiential structures, the interplay between trait empathy and lived experience, and the implications for empathy research and training.

### 4.1 From disconnection to compassion: phenomenological differences between the experiential structures

Interestingly, the four experiential structures share a common experiential foundation rooted in the connection with the actor and a subsequent peak sense of anguish. A key distinction between structures leaning toward compassion and those associated with distress is how they relate to the suffering of another. Participants who experienced disengagement (E1) reported a sense of absence, feeling detached from the primary interaction task. This detachment often involved diverting their attention from the actor to their own experiences, the actor’s context, or unrelated concerns. These findings suggest distinct embodied self-regulation strategies. As defined by Krueger (2015), embodied self-regulation refers to subject-centered aspects of our embodiment, like agency, expression, and attention, used for emotional regulation. The disconnection strategies we observed can be described as active attentional shifts and the emergence of self-centered thoughts. These strategies effectively reduce the anguish, as evidenced in our study by the decreased affective intensity or the sensation of relief, and by the sensation of a colder interaffective space. While these ways of navigating distress are effective in reducing the intensity, these experiences have also been associated with a lack of compassionate actions toward the suffering individual. For example, previous studies have shown that diverting focus to elements outside the central task, akin to a sense of not being fully present, diminishes one’s sensitivity to the pain observed in others and decreases the caring behaviors toward others (Kam et al., 2014; Jazaieri et al., 2016).

Contrastingly, the compassionate structure E4 showed a deep sense of engagement and presence during the interaction. Related to the embodied regulatory strategies in the compassionate structure, we suggest the integration of self-centered aspects of the embodiment and the search for co-regulation strategies to maintain the balance while the participants interact with the actor. Firstly, being actively present, a “mindfulness mechanism,” has been linked to reduced automatic reactivity and motor responses (Guendelman et al., 2017). Thus, relational presence has been described as a skill adept at breaking the cycle of ruminative thinking often sparked by adverse events, particularly when attuned to the suffering of others. This strategy is evident in our results as a continuous conscious return to the present moment and sensory perception of our distress without being completely absorbed in it (Guendelman et al., 2017). Secondly, embodied regulation extends beyond their own physical space allowing for a reciprocal bodily affectivity that balances affect. This suggests that embodied regulation is also embedded in the interactive dynamics. For instance, both compassionate structures show a motivation to alleviate suffering through touch or other gestures, with touch in interactive dynamics proven to reduce both personal and others’ suffering (Goldstein et al., 2018). Other gestures, like refraining from asking questions or smiling, also might help reduce distress in compassionate structures and are associated with decreased emotional intensity and a sense of warm, close interaffective space. Both factors imply that being present and possessing a willingness to alleviate suffering can effectively reduce one’s distress while maintaining engagement with another’s suffering. In our study, these categories were found to be interconnected, both in the experiential structure and the positive correlation between the presence and compassionate acts of engagements.

In summary, these extreme ways (disengagement relative to compassion) of experiencing another’s suffering reveal distinct ways of relating to others’ distress. Although both approaches are associated with reducing distress, the primary concern arises when they are used continuously. For example, more withdrawn or disengaged strategies have been associated with inhibition in engaging with individuals who are suffering (Reynolds et al., 2019). Additionally, lower levels of compassion as a trait have been linked to reduced long-term social competencies (Allemand et al., 2015). In contrast, compassion has been shown to positively impact not only the wellbeing of the compassionate caregiver but also to benefit the individual experiencing suffering, particularly within the context of healthcare (for review see Trzeciak et al., 2017). The evidence from compassion-based training helps to understand the long-term benefits of experiencing others’ suffering with compassion. Compassion-based training enhances visual attention toward others and fosters positive emotions when witnessing another’s suffering (Weng et al., 2018; Klimecki et al., 2013). It also promotes helping behaviors, such as donations (Ashar et al., 2016), while providing health benefits, including reduced stress-induced immune responses (Pace et al., 2009).

Another significant finding pertains to the differences in engagement acts between the two supportive structures (E3 and E4). Interestingly, in both structures, the primary motivation was associated with offering support through touch. Touch is known to alleviate not only the suffering of others but also one’s suffering (Goldstein et al., 2018). Furthermore, in empathy for pain, touch has been shown to increase interbrain and physiological synchronization between individuals (Goldstein et al., 2018). In our study, the intention behind alleviation revealed two subtle distinctions: the compassionate acts were either reactive anguish-oriented or balance-oriented. These differences are critical to understanding because most studies that measure the intention to alleviate suffering do not examine the driving force or quality of these bonding acts. Hence, compassionate individuals may be motivated to alleviate the suffering of others without reducing or regulating their suffering. Such compassionate strategies may be associated with traditional empathic distress and could be overlooked in other research. In conclusion, not all methods of alleviating suffering may be healthy in the long term. Anguish-driven compassionate acts, if repeated, could lead to the negative effects of empathic distress (Thirioux et al., 2016). However, this hypothesis needs further exploration in subsequent studies.

Concerning the dimensions of interaffective space, the ability to engage in an interactive setting allows for an understanding of another’s affective quality that transcends the mere lived body. As described by Fuchs (2013), the sensation of “ambiance” has been largely overlooked in emotion and empathy research. Interpersonal ambiances constitute the intermediary realm through which another’s suffering is perceived. Intriguingly, associating the affective quality of bodily resonance with these spatial affective features, particularly in supportive structures (E3 and E4), reveals an anguish characterized by an unpleasant valence juxtaposed with a warm space. These dual affective qualities are experienced concurrently and underscore the concept that compassion can encompass both positive and negative elements. According to research by Goetz and Peng (2019), both Americans and Chinese view compassion as more akin to positive emotions. Nevertheless, they report that compassionate experiences can be emotionally dichotomous, both pleasant and unpleasant. The authors suggest that positive feelings are often linked with the motivation to care for others, acts of helping, and witnessing the relief of another’s suffering (Goetz et al., 2010). Furthermore, these views of compassion have been reported to undergo changes following compassion training, where participants associated a compassionate person with more positive concepts (González-Hernández et al., 2021). Specifically, in our study, this positive affective quality was embedded within the interpersonal spaces, and participants did not report any other positive emotions such as love, caring, or peace. However, the perceived warmth of space is related to more compassionate acts of engagement, which may indicate a holistic positive emotion associated with the ambiance and an intention to alleviate suffering.

### 4.2 Empathy trait, state self-report, and phenomenological experience

In this study, trait empathy and compassion were measured using a classical empathy trait index, specifically the IRI and CS. Interestingly, specific subscales of the IRI, along with the total IRI score, were positively associated with the structures of experience. This positive correlation indicates that subjects with compassionate experiences tend to exhibit higher levels of empathic concern. This finding aligns with several studies demonstrating a relationship between empathic concern and self-reported states of empathy (Górska et al., 2023; Klimecki et al., 2014), brain–body physiology (DiGirolamo et al., 2019; Maffei et al., 2019), and interbrain coordination (Goldstein et al., 2018) However, it is important to note that empathic concern and the total IRI score did not differentiate between the two most compassionate structures (E3 and E4; please refer to Supplementary Figures S1, S2). While trait empathy is associated with lived experiences, it does not fully capture the finer elements, such as the quality of motivations behind alleviating another’s suffering. Although these scales reflect a general concern for the suffering and a willingness to help, they fall short in measuring the extent of tolerance for others’ distress and, more importantly, the quality of these compassionate acts. In this context, phenomenology offers a more nuanced approach, capturing subtleties in lived experiences through the integration of various experiential dimensions, something that standard self-report measures are difficult to capture. Examining the CS, this instrument was unable to predict the lived experience in this interactive paradigm. The CS, validated with strong psychometrics, has not been previously used to associate with the state of empathy-compassion, thus its external validity is unknown (Pommier et al., 2020). We suggest further studies to test the relationship between this scale and the state of empathy.

### 4.3 Implications of enactivism empathy research and empathy training

Understanding the nuances of graded empathy informs diverse approaches to perceiving another’s suffering, shaping future research and training.

First, incorporating phenomenology, particularly through interviews, provides a versatile tool for studying empathy from an enactive perspective. This approach offers an integrative view of empathy, emphasizing the need to merge bodily mechanisms with phenomenological experience within the experimental context to explore how these two dimensions of the body—the objective body (the physical body) and the subjective essence (the lived experience)—interact (Varela, 1996; Troncoso et al., 2023). Specifically, by examining the distinct bodily physiology within each experiential structure in this study, we can gain deeper insights into different forms of bodyssence, meaning the integration of bodily mechanisms within each experiential structure in empathic interaction (For details of this exploration see [Troncoso et al., 2024]). Simultaneously, incorporating measures from the other individual (e.g., the Actor) in future studies will allow us to investigate the co-emergence of co-bodyssence, capturing shared physiological processes and subjective experiential phenomena.

Second, developing a phenomenological self-report based on diachronic dynamics and identified experiential structures would provide a structured framework for capturing the dynamics of empathy as it unfolds in an experimental context. This would enable a more precise analysis of how subjective and intersubjective experiences evolve.

Finally, this study has implications for the foundations of empathy-compassion training and its evaluation. Given the importance of presence, emotional balance, and the quality of engagement acts, compassion training programs could use these elements as key focal points. These aspects have already been integrated into training programs based on the enactive understanding of compassion (Halifax, 2012). Moreover, phenomenology could be a valuable tool for understanding the effects of such training on these phenomenological dimensions, providing deeper insights into how individuals experience and embody compassion.

### 4.4 Strengths, limitations, and future directions

One of the key methodological advancements of this study is moving beyond the traditional picture-based and video-based empathy paradigms, adapting them to an interactive and more ecologically valid context. For example, this approach allowed us to identify phenomenological categories such as relational presence and inter-affective space, which had not been explored in our previous non-interactive studies (Martínez-Pernía et al., 2023; Troncoso et al., 2024). Although this study advances in improving ecological validity, the necessity for experimental control (e.g., structured narratives in the same environment) might obscure the dynamics of a more ecological interactive situation. Experimental control helps compare narratives for common experiences, like the anguish climax but restricts natural participant interaction. To offset this, a free interaction phase was introduced at the end of the paradigm. Future research could enhance ecological design, for instance, by simulating real-life interactions beyond the laboratory settings.

One of the strengths of assessing the temporal elements of subjective experience is the ability to detect different experiential trajectories and how they unfold over time. This approach allowed us to identify the unique aspects of each trajectory and understand the temporal complexity of experience. It highlights the value of incorporating phenomenology into experimental paradigms, as experience is often studied through self-report, which tends to overlook its diachronic dimension. The method also reveals specific gestures and acts that either nurture, support, or disconnect from the experience, and how these emerge alongside dimensions such as relational presence and inter-affective space. This highlights the multidimensional nature of experience and the interrelation of phenomenological elements. It lays the foundation for future studies to evaluate and train these gestures as integrated qualities, as exemplified in Hallifax’s training approach (2012).

Another strength and area for future work is integrating methods from various disciplines to enhance rigor, transparency, and validity. In our analysis process, we employed intersubjective validation tools, which we propose as a requirement for intersubjective validity in experimental phenomenology. Additionally, the inclusion of quantitative data reporting strengthens the qualitative richness and allows those unfamiliar with the method to better understand the results. Lastly, the use of open reporting through HTML from R, along with the codebook and interview transcriptions, provides a transparent way to share procedures and results, enabling the phenomenological community to utilize, critique, and suggest improvements.

As a limitation, the study’s sample, primarily educated, young, and healthy individuals, may not represent the broader population, and cultural factors influencing empathy remain unexplored. It is also recommended to explore diverse backgrounds, including health professionals and caregivers, and their empathy experiences. Overall, further research is needed to capture its full spectrum in more natural contexts, including longitudinal and comparative studies, cultural considerations, and incorporating neurophysiological data.

As another limitation of the sample, its size (42 participants), while adequate for phenomenological studies, is insufficient for robust correlation statistics; future studies should aim to increase the sample size to enhance the reliability of such analyses, even when using Bayesian methods.

## 5 Conclusion

Our study presents a more holistic understanding of empathy than previously acknowledged in social neuroscience and psychology. Shifting from the commonly reported binary view of empathy as distress or compassion, this study shows a more graded perspective of empathy-compassion structures of experiences, considering its interactive phenomenological dimensions and its temporal dynamics. These graded structures result in distinct approaches in interactive engagement acts, presence, and emotion co-regulation to address and resonate with the other’s suffering. Finally, these findings underscore the value of integrating phenomenology with interactive frameworks for studying empathy in more realistic settings, suggesting a more holistic approach to comprehending these complex interactive processes.

## Data Availability

The raw data supporting the conclusions of this article will be made available by the authors without undue reservation.

## Footnotes

^1^All quotations were translated literally from Spanish and slightly edited for length.

^2^For a deeper understanding of each subcategory that comprises ‘Bodily Resonance,’ including additional examples and detailed descriptions, please refer to the Codebook on Supplementary material.
