# Supplementary material for: From disconnection to compassion: a phenomenological exploration of embodied empathy in a face-to-face interaction
Source: Front Psychol. 2025 May 9;16:1522701. doi: 10.3389/fpsyg.2025.1522701 (PMC12098353; doi:10.3389/fpsyg.2025.1522701)
Supplement: Supplementary file 1 [file Data_Sheet_1.pdf]

Supplementary Material 1

1. Performance Narrative

The selected performance is as follows:

Participant: "And what is something you wouldn't want to forget?"
Actor: *Pause*... "I don't want to lose the relationship with my family, and I have children and grandchildren. What I wouldn't want to forget... [performance *with low emotional intensity]*... *Pause*... I don't want to lose my relationship with my wife. The doctor says I'm going to forget everything…I will forget everything, including the fact that Cecilia is my wife... [performance *reaches peak emotional intensity.]*... That's terrible. Because I already know that I need her. A lot. And she needs me. She needs me. *Pause*... The pain of me, losing consciousness and that she was getting lonely, and I, on my part, would also be getting lonely."

2. Analysis process

Initially, leveraging Atlas. Ti 9 qualitative data analysis software, each of the three researchers involved in the analysis meticulously examined the interviews. They began by extracting a concise overview that encapsulated the essence of each participant's described situation. Following this initial step, the researchers identified statements directly portraying the experience, termed 'meaning units'. During this process, the researchers transformed these expressions into diachronic and synchronic codes, emphasizing their intrinsic psychological significance. The definition of these codes was particularly based on the analysis of the initial ten interviews (phase 1).

After the initial coding, the researchers conducted a process of abstraction. Detailed phenomenological codes, such as body tension and body anguish, were refined into more abstract categories like body feeling. This refinement was achieved by identifying shared experiences across participants, primarily based on the analysis of the first ten interviews (phase 2). As primary categories (e.g., body resonance) and their associated subthemes (e.g., affective quality) emerged, the research team engaged in thorough discussions, fostering a unified comprehension of the main categories, subcategories, categories, and temporal phases.

The subsequent phase (phase 3) entailed the synthesis and description of the overall structure of the experience. This synthesis, built upon analyzing the first ten interviews, integrated the identified categories into a coherent structure. This structure evolved from the examination of specific elements to a fundamental understanding of the participants, achieved by systematically exploring and refining these elements. While the initial abstraction process primarily relied on the first ten interviews until reaching saturation, the refinement process continued throughout all subsequent interviews and phases.

Advancing to phase 4, two randomly selected coders from the pool of three conducted an independent subject-by-subject analysis of all the collected samples, making use of the main categories, sub-categories, and the structure of experiences established in phase 3.

Phase 5, entailed calculating the percentage of agreement in each subject. The coefficients were used throughout the analysis process to assess the level of agreement between the researchers. By calculating the level of agreement, the researchers could identify errors, omissions, and disagreements. The average of the percentage of agreement was 82%. This value indicates a high level of consensus among coders. The feedback provided in Phase 5 further aided in pinpointing and resolving any discrepancies among the researchers in Phase 6.

The final step, Phase 6, involved a collaborative review and discussion of the experiential dimensions of each participant, taking into account the level of agreement achieved in Phase 5. Through this rigorous exchange of ideas and perspectives, the researchers arrived at a consensus, enhancing the overall robustness and credibility of the study.

Throughout the procedure, our analytical approach was inherently iterative. As new categories emerged or existing ones underwent modification during the interview analysis, a thorough retrospective review of all previous analyses was performed.

3. Measures of Empathy-compassion Trait

The Interpersonal Reactivity Index (IRI) is the most widely employed tool for assessing empathy and its various dimensions (Davis, 1980). It comprises 28 items distributed across four subscales, which gauge four fundamental aspects of empathy: Perspective-Taking, Fantasy, Empathic Concern, and Personal Distress. In this study, we specifically employed the subscales of Perspective-Taking, Empathic Concern, and Personal Distress, in addition to the overall IRI score. The Chilean version of the IRI has adequate internal consistency and validity (Fernández et al., 2011).

The Compassion Scale (CS) was composed of 16 items designed to evaluate the degree of compassion that individuals feel toward others (Pommier et al., 2010; Pommier et al., al 2020). This scale encompassed four key dimensions: Kindness, Common Humanity, Indifference, and Mindfulness. Our analysis focused on the subscales of Kindness, Indifference, and Mindfulness, as well as the total CS score. The Spanish version of the CS has adequate internal consistency and validity (Pons et al. 2014).

4. Exploratory analysis of Bayesian Correlation

The following table S1 and figure S1 show the results of Bayesian correlation in terms of Kendal's tau correlation coefficient, the Bayes factors (BF), and credible intervals (CI). The variables measured were the phenomenological categories (Engagement Acts, Affective Intensity, Interaffective Space, and Relational Presence) and the structure of experience (Structure). Additionally, sub-scales and total values of IRI and CS were used.

Variable		Structure	Presence	Engagement Acts	Interaffective Space	Affective Intensity	CS Kindness	CS Mindfulness	CS Indifference	CS Common Humanity	CS Total	IRI Perspective	IRI Empathic Concern	IRI Fantasy	IRI Interpersonal distress	IRI Interpersonal reactivity total
1. Structure	Kendall's tau	—														
	BF_{+0}	—														
	Upper 95% CI	—														
	Lower 95% CI	—														
2. Presence	Kendall's tau	0.710***	—													
	BF_{+0}	$1.219\times10^{+8}$	—													
	Upper 95% CI	0.839	—													
	Lower 95% CI	0.447	—													
3. Engagement Acts	Kendall's tau	0.848***	0.646***	—												
	BF_{+0}	$3.940\times10^{+9}$	120968.601	—												
	Upper 95% CI	0.923	0.797	—												
	Lower 95% CI	0.538	0.354	—												
4. Interaffective Space	Kendall's tau	0.520***	0.627***	0.646***	—											
	BF_{+0}	3594.239	58261.655	20314.865	—											
	Upper 95% CI	0.689	0.782	0.801	—											
	Lower 95% CI	0.256	0.337	0.330	—											
5. Affective Intensity	Kendall's tau	−0.216	−0.339	−0.095	−0.050	—										
	BF_{+0}	0.084	0.063	0.168	0.190	—										
	Upper 95% CI	0.160	0.134	0.250	0.256	—										
	Lower 95% CI	0.002	0.001	0.003	0.003	—										
6. CS Kindness	Kendall's tau	0.098	0.232	0.194	0.233	0.165	—									
	BF_{+0}	0.487	2.849	1.394	2.408	0.929	—									
	Upper 95% CI	0.308	0.429	0.408	0.441	0.390	—									
	Lower 95% CI	0.008	0.034	0.020	0.030	0.014	—									
7. CS Mindfulness	Kendall's tau	0.026	0.043	−0.018	−0.053	0.269	0.561***	—								
	BF_{+0}	0.252	0.296	0.205	0.168	3.747	83821.022	—								
	Upper 95% CI	0.256	0.276	0.252	0.232	0.478	0.717	—								
	Lower 95% CI	0.004	0.005	0.004	0.003	0.042	0.313	—								
8. CS Indifference	Kendall's tau	−0.087	0.065	−0.041	−0.061	−0.021	0.293*	0.303*	—							
	BF_{+0}	0.123	0.355	0.179	0.162	0.208	11.693	14.919	—							
	Upper 95% CI	0.191	0.292	0.239	0.228	0.258	0.477	0.487	—							
	Lower 95% CI	0.002	0.006	0.003	0.003	0.004	0.074	0.082	—							
9. CS Common Humanity	Kendall's tau	−0.059	0.099	0.077	−0.093	0.102	0.429***	0.495***	0.526***	—						
	BF_{+0}	0.143	0.490	0.401	0.138	0.498	530.999	5688.680	19690.547	—						
	Upper 95% CI	0.205	0.318	0.315	0.212	0.341	0.600	0.659	0.687	—						
	Lower 95% CI	0.003	0.008	0.007	0.002	0.008	0.192	0.252	0.281	—						
10. CS Total	Kendall's tau	0.045	0.008	0.049	0.055	0.191	0.190	0.224	−0.349	0.118	—					
	BF_{+0}	0.293	0.228	0.319	0.335	1.256	1.618	2.881	0.049	0.609	—					
	Upper 95% CI	0.269	0.253	0.294	0.299	0.411	0.385	0.415	0.111	0.324	—					
	Lower 95% CI	0.005	0.004	0.005	0.006	0.018	0.022	0.034	0.001	0.009	—					
11. IRI Perspective	Kendall's tau	0.145	−0.029	0.112	0.101	−0.085	0.118	0.138	−0.200	−0.044	0.268	—				
	BF_{+0}	0.842	0.178	0.546	0.494	0.150	0.606	0.775	0.076	0.156	6.768	—				
	Upper 95% CI	0.346	0.231	0.340	0.332	0.224	0.324	0.340	0.147	0.213	0.455	—				
	Lower 95% CI	0.013	0.003	0.009	0.008	0.003	0.009	0.012	0.001	0.003	0.057	—				
12. IRI Empathic Concern	Kendall's tau	0.384***	0.187	0.206	0.246	0.009	0.092	0.146	−0.294	−0.213	0.278	0.267	—			
	BF_{+0}	130.245	1.414	1.630	2.930	0.249	0.457	0.860	0.057	0.073	8.380	6.635	—			
	Upper 95% CI	0.560	0.390	0.418	0.452	0.276	0.303	0.347	0.122	0.143	0.464	0.454	—			
	Lower 95% CI	0.151	0.020	0.022	0.035	0.004	0.007	0.013	0.001	0.001	0.063	0.056	—			
13. IRI Fantasy	Kendall's tau	0.200	0.040	0.239	0.258	−0.045	0.159	0.018	−0.148	−0.096	0.204	0.273	0.421***	—		
	BF_{+0}	1.890	0.288	2.636	3.549	0.182	1.025	0.236	0.093	0.118	2.036	7.517	419.571	—		
	Upper 95% CI	0.393	0.274	0.446	0.462	0.245	0.358	0.250	0.165	0.187	0.397	0.459	0.594	—		
	Lower 95% CI	0.025	0.005	0.032	0.040	0.003	0.015	0.004	0.002	0.002	0.026	0.060	0.185	—		
14. IRI Interpersonal distress	Kendall's tau	0.141	0.168	0.211	0.177	0.115	0.113	0.043	−0.050	−0.036	0.026	0.036	0.077	0.087	—	
	BF_{+0}	0.805	1.085	1.738	1.112	0.560	0.575	0.288	0.150	0.163	0.251	0.272	0.394	0.432	—	
	Upper 95% CI	0.343	0.373	0.422	0.393	0.350	0.320	0.267	0.210	0.217	0.255	0.262	0.292	0.299	—	
	Lower 95% CI	0.012	0.016	0.024	0.016	0.009	0.009	0.005	0.003	0.003	0.004	0.005	0.006	0.007	—	
15. IRI Interpersonal reactivity total	Kendall's tau	0.293*	0.113	0.281	0.322*	0.018	0.195	0.127	−0.232	−0.122	0.298*	0.443***	0.569***	0.594***	0.380***	—
	BF_{+0}	11.709	0.566	5.330	11.594	0.263	1.759	0.674	0.068	0.104	13.101	855.318	120000.325	368893.987	115.985	—
	Upper 95% CI	0.477	0.329	0.483	0.520	0.282	0.389	0.331	0.138	0.176	0.482	0.613	0.724	0.746	0.557	—
	Lower 95% CI	0.074	0.009	0.052	0.078	0.004	0.023	0.010	0.001	0.002	0.078	0.205	0.320	0.343	0.147	—

Note. For all tests, the alternative hypothesis specifies that the correlation is positive.

* $BF_{+0} > 10$, ** $BF_{+0} > 30$, *** $BF_{+0} > 100$

Table S1. Bayesian Kendall correlation between phenomenological categories, experiential structures, and the various subscales of the Interpersonal Reactivity Index (IRI) and Compassion Scale (CS) are shown in the table. Supported correlations are shown with an asterisk (*). *Bayes Factor > 10, *** Bayes Factor >100

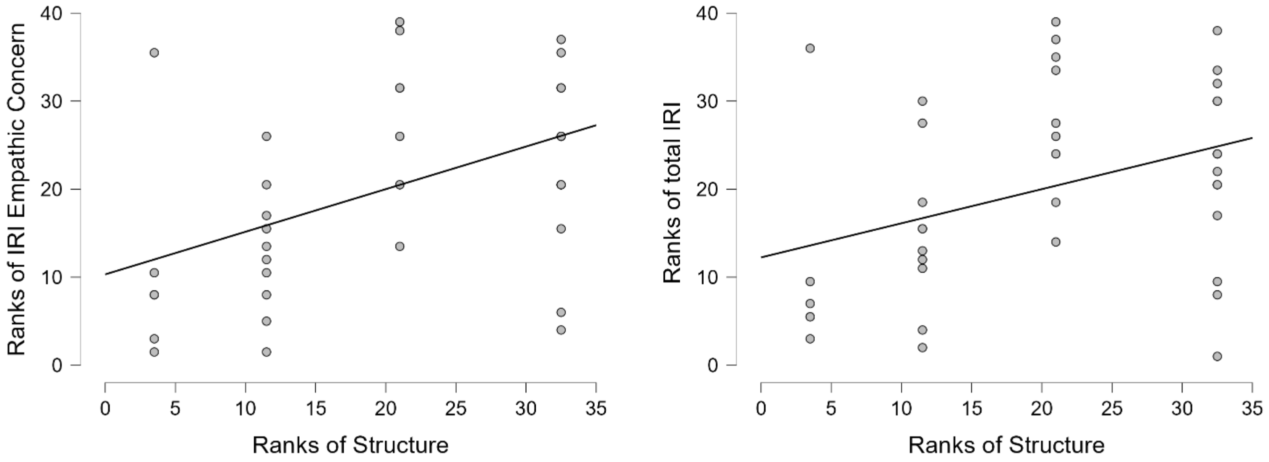

Figure S1. *Scatter plots illustrate the relationship between the structure of experience (x-axis) and two empathy-related measures: empathic concern (left plot) and total IRI score (right plot) on the y-axis.* Each point represents individual data, with a trendline indicating the overall direction of the relationship between the structure of experience and each empathy measure.

5. Examples of Interview

Here are two examples of phenomenological interviews. Some comments are highlighted in red to explain certain decisions and to suggest alternative interviewer interventions that could have improved the exchanges. The interview was translated into English. “I” represents the interviewer and “P” represents the participant. In some interviews, the audio was started after the description of the study's objective, the interview, and some initial instructions.

Example 1.

I:

The goal of the phenomenological interview is to capture the experience exactly as you lived it. The idea is to describe it in terms of bodily sensations, emotions, and thoughts that arose, rather than judging the experience. For example, you might describe the feeling of joy, like a sensation in your chest that opens up, and how you feel your face smile and expand. That would be a good phenomenological description. However, when we begin to judge the experience, for example in terms of whether it was good or bad, that would be a judgment of the experience. So in everyday life we generally approach experiences with judgment, that is, we judge how good or bad the experience is rather than describing in depth what the experience is like. The questions will be directed to certain bodily sensations, and emotions, among other dimensions and the idea is to approach those experiences with curiosity, and openness.

I:
If I invite you to close your eyes… I would like you to make yourself as comfortable as possible. May I ask you to bring your body and mind back to the last interaction with Mr. Marcos, when he said, 'I don't want to forget…'? The idea is for you to walk through what you experienced from the moment he entered until he left. When you are ready, just let me know.
P:
I'll let you know.

I:
Tell me what happened during this last interaction.

P:
Mmm... When Marcos came in, I felt... something. I don't know how to explain it... It was like a feeling I couldn't quite define. I just knew that I didn't like what I was seeing, I didn't like what was happening. Not necessarily the situation itself, but rather something about it ….I felt like maybe I personally usually shy away from this kind of experience, but like I wasn't comfortable with having a situation as helpless as the one he was having, I mean, especially when he told me that he already knew that he was going to forget things. And that he didn't want to do it, but still no, he couldn't do anything about it. He there said like the repercussions that it had at the end I was pretty much... Eh. I don't know because maybe I must have the feeling like lending a helping hand and when, like when it puts me in this context like I feel like I can't do anything to help, if I even started like looking like at the door like please, like that already. Please, this question was not the best of all and it was... It gave me that feeling.

I:
And that feeling, uh... So he comes in and you had these feelings of ... Like unpleasant, like being exposed, it was not a comfortable situation at all.

P:
Aja [sound to indicate understanding]

I:
Then he starts his story, right? What did you feel like on a bodily level?

P:
In the story. I was feeling. As I went on, I felt as if I wanted to be as abstracted as possible, that is, in general, whenever I am with a person, I am always attentive to what he/she tells me and how he/she behaves to understand him/her beyond what he/she tells me. And, in this case, it was a moment in which I started to dissociate, so to speak, as I no longer wanted to be present in that interaction, as I did not want to be... I didn't want to continue communicating with that person, because what he was telling me as if it was not him.... was generating a feeling in me. As I became aware of that connection forming, I could feel that reaction building up inside me, and before I even made a conscious decision, I had already started to withdraw.
I:
And that sensation you say there, how did you feel it in the body?

P:
Like, like something like a campfire, so to speak like something well. Not like angry but like something impotent that kind of directs, like not anger, but like... the impotence kind of generates something like it triggers like that feeling that I don't want to be there anymore. It made me uncomfortable, uncomfortable, contextually that.

I:
And that…, at what point did that happen?

P:
That happened when he started talking to me about how he was going…., he didn't want to forget his wife.

I:
Where did you feel this campfire?

P:
So here. Feeling like helplessness underneath the [Positions hands-on Chest-Abdomen to show him].

I:
Between the chest and the abdomen?

P:
I would say yes, that is to say…I don't remember much because as I say when I'm in that moment, like disconnection, I kind of try to just comment, to abstract myself from everything I'm feeling at that moment.

I:
I will ask you to reconnect with the moment when he tells you that he doesn't want to forget Cecilia, his wife. How did you feel those sensations at that moment?

P:
Yes

I:
That one.

P:
Like something like hard [Hands on Chest-Abdomen], like something tense like that.

I:
Did you feel it here [Hands on Chest-Abdomen]?

P:
I felt it much more in my legs, my legs were too tense. In fact, all of a sudden, like I was trying to loosen up a little bit, and out of nowhere, I was like, hey, I don't know when I must have

tensed them so much. I was there... I felt them very tense, like I had a very strict stop, so to speak. [Here the interviewer could have elaborated on how he did to loosen up].

I:
In this interaction.

P
Yes.

I:
And after he tells you about his wife, what happens?

P:
Um... I started thinking about... I don't know why it happened, I mean, I guess I can also say this in the interview, but I had a grandmother that I didn't know very well because, I mean, that's exactly why I didn't know him. When she was like that she already had Alzheimer's, she had developed Alzheimer's disease. And I remember that there were very few times that my dad would go to see her, and he would tell me if I wanted to see her, but I didn't have any emotional bond with her. I don't know why I started to think about that, as if she never had that role for me, I mean, the only thing I remember, the memory I have of her is that once I told my dad, insisting, as if I could tell him, please, that she had Alzheimer's, because obviously she forgot and I was very young, and my dad told me very well, tell her, but no..... So like no, I didn't listen and I kind of still insisted and kind of told her like that, like to at least see what her reaction would be like. And that's when I realized that Alzheimer's didn't have this kind of behavior of, I mean, forgetting, but it doesn't have this kind of hydroponic behavior either, it doesn't exist like that state. So, I don't know why I remembered the context of that specific moment when he started talking to me about not wanting to forget his wife.

I:
Then, if I understand correctly after he tells you about his wife, these thoughts begin.

P
Of course, more personal thoughts.

I:
Were these thoughts accompanied by anything else? I

P:
It was accompanied by a picture of my grandmother.

I:
How was the speed of those thoughts and Images?
P:
It was fast, everything was like ephemeral in my thoughts and nothing was like to stay long like that.

[Here we could have gone deeper into these images, but due to the objective of the study we prefer not to go so deep into this]

I:
I would like to take up those that although, I understood you felt a need to abstract yourself from the interaction. You want to get out that you didn't want to be in the situation, at what point does that happen?

P:
It happens exactly at the moment in which... It's just that since you asked me the question I said that I had an idea like that and I.... I think it was exactly at the moment when he started talking about how he didn't want to, he didn't want to forget his wife, but because they were going to be alone, when he said that they were going to be alone, I was shocked, like he was going to forget, they were going to be alone and there was nothing to do to change that, that's when...

I:
How did you feel at the time?

P:
Emm, I felt uncomfortable, obviously, because I knew that I kind of already... I didn't know like when they were going to open the door and it was going to be over like the interaction. And I was waiting for it to happen somehow. I mean, clearly not That question didn't make me comfortable, I felt like it was super tense. That whole moment, I was clear with that kind of defense, in which I didn't want to continue, I was disconnecting because if I continued connecting I was going to continue generating that motion, identifying it. So, I kind of unconsciously made the decision not to continue valuing the interaction of that question.

I:
And how did you make that decision?

P:
I looked at the floor, I looked at the floor, I stopped looking at him. I cut that connection like that. Not disrespectfully, but. Eh. Not empathetically either, so to speak.

I:
So, at that moment uh you have your thoughts and you look down at the floor. And what happened in the body?

P:
In the chest, I felt a lot like. Look, it probably comes ...., probably because when I am in that context, the same that happens to me when I am in tests - that state I associate it a lot with that so sometimes I don't realize it because I am too abstracted, so to speak, but indeed I am restless and I realize it by how all my muscles are tense, that is, what happens in my legs is something more like I can't, like I can't move from the place, but it's like what is here. It's like everything I eat is the emotional part, although it is reflected in this part, yes. [Here, there could have been much more depth regarding the sensation in the chest. For example, how intense was it? How large was it? How dense did it feel?]

I:
How intense was that feeling in the chest?

P:
At the beginning, not so much. In fact, there it started to generate when it was the biggest and that was when she was already looking at the ground. I was looking at the floor and that was the pick of that behavior.

I:
And this space between you and two Marcos, how did you experience it, is there any other characteristic of this place of interaction? [here it would have been convenient to ask only one question at a time]

P:
Are you asking if I felt something in the atmosphere?

I:
Not like, for example, when I fight with my family there is a kind of tension in the environment.

P:
Ya.

I:
In this atmosphere, how did you feel it?

P:
I mean, of course, I feel that that last specific question, as if he took us out of the experimental context and kind of put himself in a personal context as if it was already real what was happening and so much so that I noticed it as if... Saying those things out loud is different than always thinking it, so when he says them out loud like you're still in that icy air in the room, and in the end it's super Eh shocking to think that effectively, although not physically, you're both going to be alone eventually, so in the end Having that present and telling him, and like it's like something that comes with time and waiting for that event is super Em dense.

I:
So while I understand there you felt like an icy air in the room?

P:
Yes.

I:
How did you feel that cold air?

P:
More than a coldness, it was more like the concept that I wanted to refer to is that I felt like a context like I don't know, as if. Like now... like relaxation, like let's say that you feel the air like

that, so to speak, and then it's not like that anymore like everything is like an as if the piece was a tightened thread like that.

I:
And at that moment, how did you feel the closeness?

[All these questions were to be able to rescue the interaffective space]

P:
I felt close at the end because, in the whole experience, emotions were involved, somehow that's why I feel close to the other person, more than anything else.

I:
And in this last one?

P:
In this last. Even though it was more negative, but still, the fact that I was like more abstract made it less. Because I couldn't kind of give feedback on those emotions as I would have liked.

P:
Right because in the end if I stayed in the interaction, that feeling grew and grew and grew and grew. If I disconnected, I left it there, but it still starts like the countdown, which is when I am abstracted from this environment that is waiting for me, waiting for me to generate that feeling. It's like the first defense mechanism, so to speak. In fact, later, when you left to do something, I was left here alone. And I don't know why I started to think like that when this experiment was over, like that feeling that I wanted to leave like that. And it was weird, I mean, from there I thought to myself, I said, like, “Wow!

I:
And did you feel that feeling of wanting to leave in your body like that?

P:
I mean, I felt like I was, like I was starting to do that process, in which one evaluates what stops me from leaving like that, what stops me from grabbing my things and leaving like that. I mean, even if it is hypothetical, in general, I feel that I usually make that kind of decision, I mean, obviously, in the academic context I have a different behavior, much more professional, so to speak, academic, at the end I am here on my own and I come here to face this objective calmly. But in the context of, for example, the friendly ones with friends, I don't like someone that I don't like, I fish my things and I leave, not something like to imply a negative message, I just feel that I don't have to submit myself to these environments in which I am interfered with in a way that I don't like to do, it sounds like something too quico so to speak, that doesn't do me good, I would say, because this is what I mean, that is, some people have more, eh? Resistance like this kind of emotion and as much as I feel that I can behave professionally, I marked the interview. I consider myself very sensitive, even if I can behave differently, I feel things a lot, so for example, as soon as he arrived at Two Frames I instantly connected with him. So when he makes me feel good things, I feel it very well and I like it, but when you are going to feel bad things, it is very hard for me, like not feeling it, so what is going to follow? In

general, I think that's why I have like this... like looking at the floor like this because it's like that. For me, it's like it's better to give like that, like a message, like compassion and empathy. Yeah, when I usually do that, like a lot of times I don't make it as real as it is, even as real as the other person maybe never conceived it. I mean, it could become worse. So that's been my experience from a very young age usually. [Here we could have rescued some elements, although it was more satellite information than pre-reflective experience. Due to the time elapsed, the interview is closed with a last question].

I:

And after he leaves, what happens?

P:

Total distension. So I knew it was the last question, but the first thing I took my head in I was like calmer, calmer. Other than that I realized that being. Connecting with another person is not the same, that is, I felt I had to use more resources than usual, to generate that connection, that bridge. Although I'm very experiential, I don't deny myself this experience, it's not that I don't enjoy it. Obviously, because of the questions I had to ask him, the experience was different, but I kind of like people who have something to talk about in general, so to speak. [Given that the interview only focuses on the interaction and not when leaving, I do not know of any interviews that delved into this, but it could be an important phase to explore in other studies]

I:

Well, that is the phenomenological interview, If we see what you really felt, it kind of opens up a lot. Hey, your experience. Thank you very much for participating, do you think your interview captured your experience?

P:

Yes

I:

Anything else you would like to add?

P:

No

I:

Thank you very much for your participation

Example 2:

I:

The goal of the interview is for you to describe how your experience was—just as it was in the last interaction. Only the last one. Let me explain some details: the idea is to approach it with curiosity and openness, to see what happens in your body, emotions, thoughts, and attention. I will guide you through describing the experience exactly as it is, without judging it. For example, a judgment might simply label the experience as good or bad. In this interview, I will help you find the experience at the bodily level and see how it fluctuates over time. Could you tell me about the experience you had in this last interaction? By "experience," I mean bodily sensations, emotions, thoughts, changes, and the attentional focus you had during that interaction. For this, if you agree, I will ask you to close your eyes. Make yourself

comfortable—perhaps use a cushion if needed— and recall in your body and mind the last interaction you had with Don Marcos.

Think back to when you asked him what he didn't want to forget, and he told you about his wife, his son, and his grandson. Once you're ready, you can open your eyes and begin.

What did you notice happened during this last experience?
P:
I felt kind of sad.
I:
Um.
P:
More than feeling sad for him, I felt sad for myself, in part, because these are things that could also happen to me.
I:
Mhm. And was that sadness constant over time, or was there a particular moment that made you feel sad?
P:
Uh, well, it was present throughout, but the moment I felt the most sadness was when he mentioned his wife.
I:
When he said he didn't want to forget his wife. And after that moment, when he mentioned his wife, what happened?
P:
Well, I listened to him. He said he was afraid and that he didn't want to leave her alone. And I also related that to my own experience, my own life. And I understood that feeling.
I:
Mhm. And before that peak moment—when he mentioned his wife—what was happening? From the moment you asked him and he started telling you until he got to his wife?
P:
Yes. Uh, what I did most was observe him. I noticed how his attitude changed, how he hugged himself, because... well, it was a rather emotionally intense question. Mhm. So I started observing him and had this reflex, this need to approach him, but I didn't. Or at least, I wanted to hold his hands because he was shaking a lot and fidgeting with them.
I:
And was that sadness already there?
P:
Uh, yes. Yes, but more than anything, it was this feeling of wanting to hug him and console him, to contain him. More than anything.
I:
What was that sadness like?
P:
It was a sadness that came and went throughout the interaction we had. And in this last moment, yes, it was there, but in part, that was what drove me to want to hug him.
I:
And what happened with that impulse to hug him?
P:
In the end, I did want to hug him, or at least hold his hands.

I:
Going back to the feeling of sadness, where in your body did you feel it?
P:
In my chest.
I:
In your chest, here? And what did you feel in your chest?
P:
Like... I don't know how to describe it. Like a pressure [Hands on the Chest]. But out of anguish, out of sadness.
I:
A feeling of pressure here? [Hand on the chest]
P:
Yes.
I:
And how intense was it?
P:
At that peak moment, it was strong.
I:
Very strong?
P:
Yes.
I:
And how did your whole body feel at that moment?
P:
My hands were tense, but because of that urge to get closer and hold him. Uh, and well, the pressure in my chest... In my legs, I don't remember any specific feeling. More than anything, I was still.
I:
So, if I understood correctly, at that moment, you also had the urge to get closer to him?
P:
Yes.
I:
And how did you experience that urge to get closer?
P:
Uh, I thought about it, and at one point, I moved my arm, but I put it down right away. I wanted to sit next to him and talk, to have that conversation while sitting together. More naturally and closer
.
I:
Closer. Okay. And when did that happen, what you're describing?
P:
Uh, uh, for most of the time, honestly. That urge to sit with him and talk. But more after the peak moment.
I:
After this intense moment, what happened with the sensations you mentioned?
P:
Uh, they diminished as we kept talking. In the end, we talked about how life is, that there's not much one can do. That's when the sadness eased a bit, but it was still there.

I:
Was that from the moment he mentioned his wife until the interaction ended, or until he finished answering your question?
P:
Until the interaction ended.
I:
And from when he finished answering your question, how was the sadness?
P:
It decreased compared to the peak moment.
I:
What changed in your experience as that sadness decreased? [Or what did you do to make it decrease?]
P:
Well... Uh. Uh, to bring it down, I started thinking about what to say to him so he wouldn't dwell on it too much because his gaze dropped—he was deep in thought about it. So I thought about what to say to shift his focus. And naturally, that helped reduce the sensation in my chest.[The interview is briefly interrupted as the interviewer notices the interviewee needs a moment]

I:
Mhm. Earlier, you mentioned that you also related this to your own experience. If you'd like, you can have some water—I'll get you something.
P:
Ah, uh-huh, yes.
I:
Can we go back to when you said you connected it to your own experience?
P:
Yes, I meant that... I put myself in his place, but instead of thinking about his wife, I thought about my partner, his children, and my family. So, that's what I meant by relating it to my own experience.
I:
And when did that happen?
P:
Um, mostly when he started talking about the people he didn't want to forget.
I:
His grandson, his son. And later, when he mentioned his wife, what happened with those thoughts?
P:
They remained because he talked about his family, and I thought about mine. Then he mentioned his wife, and I thought about my partner. And after that, I kind of forgot about it because I was focused on figuring out what to say to shift his attention.
I:
How did those thoughts emerge—did they come as inner dialogue or as images? [Here the question could be modified to "What are the characteristics of this thought? Or what are these thoughts like?]
P:
Images—of my family, my partner.
I:
How fast were those thoughts and images?

P:
Not too fast—somewhere in between. They were present while he spoke, but I was still paying attention to him. It was like his words evoked those thoughts in me as he talked.
I:
How did you experience being present in that moment?"
P:
I mean, I drifted a bit, but other thoughts were crossing my mind while I listened. Still, I was paying close attention to him—what he was saying, his expressions, his movements, his gestures.
I:
At the specific moment when he mentioned his wife, how was your attention?
P:
Mhm. It was divided and shared between his story and my thoughts. But I was still very focused on him—on his gestures, on how thinking about her was reflected in his movements, his gaze.
I:
How did you feel the space between you and him?
P:
Mhm. It wasn't uncomfortable, but there wasn't exactly a close connection either. He was still someone outside my circle, so that boundary was there. But even so, his words and experiences impacted me.
I:
And in the rest of the conversation, when he talked about going to the beach or his childhood, how did you feel? [here to change the focus of the experience if it brings to the positive experience so that the participant is not left with that negative feeling].
P:
Um. It was... nice, like a warm moment, remembering those things. He spoke warmly about his family.
I:
That would be all. Thank you so much for your participation.
I:
Do you feel that this interview reflects your lived experience in the interaction with Don Marcos?
P:
Yes.
I:
Would you like to add anything else?
P:
No.
I:
That would be all. Thank you so much.

References

1. Davis M. H. (1983) Measuring individual differences in empathy: evidence for a multidimensional approach. J. Pers. Soc. Psychol. 44, 113–126
2. Fernández, A. M., Dufey, M., & Kramp, U. (2011). Testing the psychometric properties of the Interpersonal Reactivity Index (IRI) in Chile. *European Journal of Psychological Assessment*.
3. Pommier, E., Neff, K. D. & Tóth-Király, I. (2020). The Development and Validation of the Compassion Scale. *Assessment* **27**, 21–39
4. Pommier, E. A. (2010). The compassion scale. Dissertation Abstracts International: Section B: The Sciences and Engineering, 72(4-B), 2697
5. Pons, G. B. (2014). Cultivating healthy minds and open hearts: A mixed-method controlled study on the psychological and relational effects of Compassion Cultivation Training in Chile. *Institute of Transpersonal PsychologyProQuest Dissertations Publishing, Degree Year* **2014**, 3631418
