# Supplementary material for: From disconnection to compassion: a phenomenological exploration of embodied empathy in a face-to-face interaction
Source: Front Psychol. 2025 May 9;16:1522701. doi: 10.3389/fpsyg.2025.1522701 (PMC12098353; doi:10.3389/fpsyg.2025.1522701)
Supplement: Supplementary file 2 [file Data_Sheet_2.pdf]

Supplementary Material S2: CODEBOOK

The following codebook presents and describes the four main categories that emerged from the empathic experience shared between the participants and the simulated Alzheimer's patient, portrayed by an actor (Mr. Marcos), during a semi-structured interaction. The four emerging categories were bodily resonance, relational presence, interaffective space, and dis/engagement acts. Additionally, the main subcategories that make up each category are described.

This codebook was created by considering some of the key contributions from Mihas (2019) on how to structure this type of material. Each main category includes a description, its importance, an example, and a reflection. The description provides information on the application of the code in the analysis, while the importance offers insight into the role of the code in the research. The example is a textual quotation illustrating the specified code. The reflection explains the process of code transformation during the analysis or its relationship to other codes.

Moreover, before addressing each main category, a diagram is included to visualize the relationships between the main categories and their subcategories.

1. MAIN CATEGORY: BODILY RESONANCE

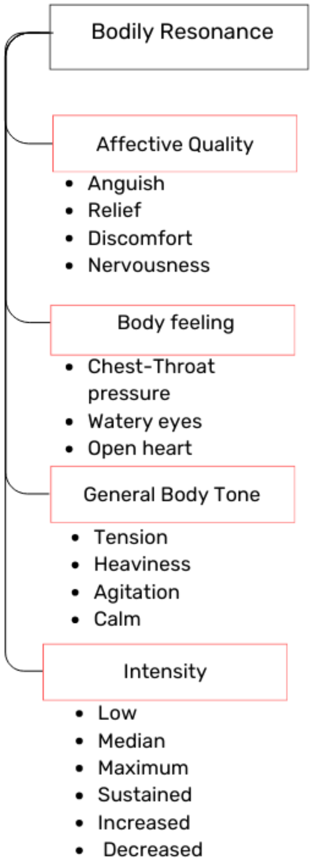

Description: Bodily resonance encompasses all codes and statements that describe the participant's bodily involvement in synchrony with the suffering of the actor. This main category includes the following subcategories: affective quality, body feeling, general body tone and intensity. Specifically, it focuses on how participants, throughout the interaction, genuinely feel the other person's suffering within their bodies. This connection is expressed through diverse bodily-affective perceptions, including localized pressure in the chest and throat, as well as an overarching sense of bodily tension.

Importance: The role of this main category is to holistically capture the corporal-affective experience of the participant when engaging with the suffering of another person.

For example:

[Referring to the moment when the actor says he does not want to forget his wife] *“It feels like it's being squeezed. And my body feels heavy. So, like, what? I had a hard time*

standing upright. I feel like my body is heavy, my throat is a little bit tight, and my eyes, like, they're, like, tears are coming out. It's like a tickle that gives—I don't know how to describe it—but it's like a sensation in my eyes." (S05)

Reflection: This central category has been previously used by this research group in other studies on empathy for pain. As such, it emerged as one of the most relevant categories of analysis during the first stage of phenomenological analysis.

1.1 Subcategory: Affective Quality

Description: Participants' Affective Quality refers to their physical-emotional sensations or affective tone, which aligns with the narrative and experiences of the actor. The most central and frequently emerging affective qualities were anguish and relief. All of these manifested in harmony with what the actor was narrating.

Importance: This subcategory explores the participant's affective response in an integrative way, capturing the corporeal-affective root of the experience. It includes specific affective tones, such as distress, relief, and tension, providing a detailed classification of emotional responses and enriching the phenomenological description of interacting with another person who is suffering.

For example:
[Referring to the moment when the actor says he does not want to forget his wife] "*Emm. When he started talking, it was normal until he mentioned Cecilia. I don't know why, but I felt distressed... I felt like crying." (S24)*

[Referring to final moments of the interaction] *"The chest pain went away a little, but what bothered me the most was the pain in my throat. Then it passed. When it was over, everything passed." (S09)*

[Referring to the initial moments of the interaction] *"I was kind of expectant or like waiting for him to say something that was like... I was kind of tense, waiting for him to say something that was going to trigger me, like grief or anguish, because I knew what the question was going to be about. So, I was kind of listening very carefully to what he was going to say and how to prepare myself for anything that might trigger grief, to keep me on my feet." (S19)*

Reflection: This subcategory emerged during the second phase of the analysis process. Its early emergence highlights the relevance of emotional responses in the participants' empathic experience within the context of the study.

1.2 Subcategory: Body Feeling

Description: This subcategory encompasses statements where participants describe their internal bodily sensation that emerge in synchrony with the events narrated by the actor. Most participants referred to sensations of pressure in the chest and watery eyes. These sensations were associated with sadness and anguish, varied throughout the interaction, and were anchored to the moments of the actor's greatest suffering.

Importance: This sub-category deepens into participants' descriptions of their internal and external bodily states that synchronize with the narrative events shared by the actor. The lived bodily sensations, such as pressure in the chest and watery eyes, serve as tangible manifestations of the empathic connection.

For example:

[Referring to the moment when the actor says he does not want to forget his wife] *“Like during he was talking a little bit about the lady, when he started talking about the lady, of course, it's like sad, but when he says he's going to leave her alone it was like a lot of pain in my chest, feeling tenser and everything.”* (S04)

[Referring to the moment when the actor says he does not want to forget his wife] *“My eyes started to fill with tears because it is difficult for your husband, your wife, your children to go through this thing that Alzheimer's, which is a very complicated disease. And you get to forget everything, your whole life, the person you are with, horrible. Horrible, I felt like crying, my eyes filled up with tears..”* (S09)

Reflection: This code emerges from descriptions of internal states, such as pressure in the throat or watery eyes. Once established, this category remains consistent without further transformation.

1.3 Subcategory: General body tone

Description: This subcategory includes statements where participants describe a general bodily state that pervades their entire experience during the interaction. Unlike localized sensations, these bodily experiences extend beyond specific areas and encompass a more diffuse, overall feeling. Participants often referred to sensations such as tension, heaviness, agitation, and calm.
Importance: This sub-category provides insight into the broader bodily experiences that synchronize with the narrative events. These sensations reflect a holistic embodied response rather than discrete localized feelings, offering a more comprehensive understanding of how bodily states accompany empathic engagement.

For example:
[At the beginning of interaction] "*The anguish...in the whole body. Yes, in the whole body, because at that moment I remember that I felt, I mean I felt like vibrations in my body*" (S2)

Reflection: This code arises from descriptions of generalized bodily states, such as general bodily tension. Once identified, this category remains stable and other specific elements are added as the analysis progresses (e.g. agitation).

1.4 Subcategory: Intensity

Description: This subcategory refers to the intensity of the bodily-affective sensations experienced by participants. It encompasses the degree to which the affect is maintained at its maximum intensity or diminishes. In other words, it explores how participants experience the depth of their bodily and affective sensations and how these emotional intensities fluctuate throughout the interaction, responding to the affect they perceive in response to the affect they perceive in the other person.
Importance: The subcategory "Intensity" within the main category "Bodily Resonance" is crucial for capturing the dynamic and fluctuating nature of emotional experiences during empathic interactions.

For example:

[Referring to the moment when the actor says he does not want to forget his wife] *"At the beginning, when he talked about his wife, saying she would eventually leave him alone, it felt stronger and stronger. It gave me more anguish; in the end, my chest tightened, and my throat closed up too." (S22)*

[Referring to the moment when the actor says he does not want to forget his wife] *"It was building up slowly until he said, 'No, but I really don't want to forget,' and then it shot up." (S28)*

[Referring to the experience in general] *“Yes, it varied. As I was saying, when he told me about his wife staying alone, that was a strong sensation. Then, it kept going up and down, but the revelation came from that interaction.” (S3)*

Reflection: This subcategory initially emerged as the "maximum" and "sustained" codes. However, throughout the analysis, additional codes were included to reveal further nuances in the intensity and emotional fluctuations experienced by participants when interacting with the actor.

2. MAIN CATEGORY: INTERPERSONAL PRESENCE

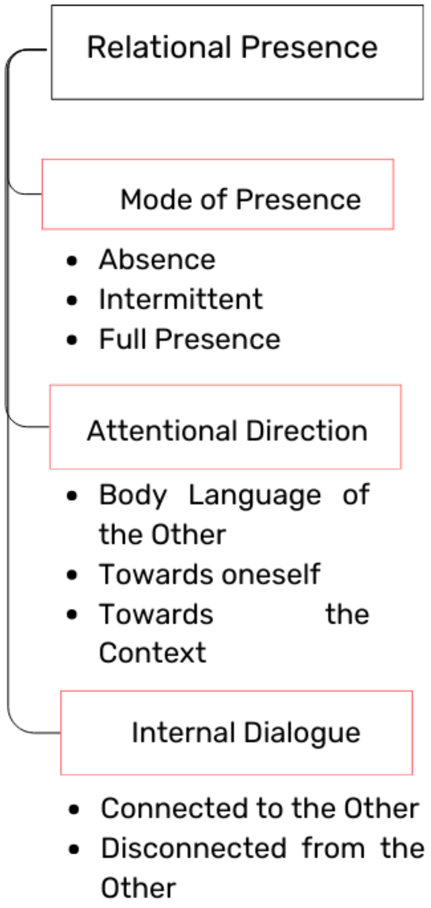

Description: During the interaction, participants experienced nuanced and dynamic states of relational presence, reflecting the extent to which they were fully engaged and connected to the unfolding moments. The spectrum of relational presence includes various states, ranging from partial distancing or a sense of absence to deep immersion and unwavering attentiveness. These different qualities of presence are shaped by two fundamental aspects: the focal point of attention and the internal dialogue that emerged in each participant, moment by moment. Three forms of relational presence emerged as a gradient: absence, intermittent, and full presence.

Importance: The main category "Relational Presence" provides critical insights into the varying degrees of engagement and connection experienced by participants. It offers a holistic perspective on the interpersonal dimensions of empathy.

For example:

[Referring to the moment when the actor says he does not want to forget his wife] *"I paid attention to myself. When I wanted to understand more, I looked into his eyes. That I feel that in turn as honesty first and foremost, then me. And all the time, I looked at him in the eyes. I can't explain it. That's why I started to feel sad—anguish, even. I got a lump in my throat..."* (S9)

Reflection: This central category emerged and evolved from the coders' reflections during phase two of the analysis, following the identification of elements related to attentional focus and internal dialogue during the interaction. The quality and intention behind these elements shaped the participants' relational presence.

2.1 Subcategory: Absence

Description: It refers to a form of presence that arises as a sense of being disconnected from the interaction, with attention directed elsewhere, beyond the focus of the person one is in contact with. In addition, internal dialogues tend to stray from the present moment, often wandering towards thoughts about personal family matters or the context of the actor. This particular state of presence reflects moments when participants experience a partial disconnection, either temporarily or more prolonged, characterized by scattered attention and limited immersion in the shared experience.

Importance: This subcategory characterizes and describes how the experience of being absent in an interaction emerges. The codes within this subcategory provide nuanced insights that enrich the understanding of the most disconnected experiential structures in empathic interactions.

For example:

[Referring to the moment when the actor says he does not want to forget his wife] *"Mr Marcos, in one way or another, guided me as if he were a voice-over. But my presence shifted into a kind of review of my life—my body, my attention, in another space. Another space. No, not here, not here, not here. Less... Less attentive to this place and the dialogue."* (S26)

Reflection: This subcategory emerged during the second phase of the analysis process. The "absent relational presence" subcategory consolidates codes that characterize moments of disconnection from the interaction.

2.2 Subcategory: Intermittent

Description: Intermittent interpersonal presence manifests as a continuous oscillation between being absent and fully engaged. Participants vividly describe a constant ebb and flow, alternately immersing themselves in the actor's experience and redirecting their focus to other elements. This subcategory captures the dynamic and fluctuating nature of participants' engagement, highlighting the interplay between moments of complete absorption in the interaction and intervals marked by partial withdrawal or redirection of attention.

Importance: The subcategory "Intermittent interpersonal presence" offers a nuanced perspective on relational presence by emphasizing the continuous oscillation in participants' engagement. It underscores the complexity of empathic experience, showing how individuals navigate between different states of attention and emotional involvement throughout interactions with those who are suffering.

For example:

[Referring to the moment when the actor says he does not want to forget his wife] *"I was kind of listening to his story, but at the same time, at one point, I looked away, trying to process the emotion a little bit as well."* (S11)

Reflection: This code arises from the need to depict the fluctuation of relational presence. It emerged during the second phase of analysis when analysts identified participants who experienced presence with nuances and variability.

2.3 Subcategory: Full Presence

Description: In the full presence dimension, participants become immersed in the actor's narrative, attentive to the actor's bodily expressions. This heightened presence becomes evident as participants delve into intricate details of the actor's facial expressions, hand gestures, and posture. Active listening becomes a hallmark, characterized by minimal interruption of internal dialogue. Participants invest their full attention and awareness in the unfolding interaction in moments of full presence.

Importance: This subcategory is crucial for representing the highest level of engagement and attention during empathic interactions. It provides valuable insight into the depth of the connection between the participants and the actor, enriching the understanding of relational dynamics and the empathic experience.

For example:

[Referring to the moment when the actor says he does not want to forget his wife] *"I think that if thoughts could take the form of images...¿how could I make his life if I forgot something so essential to him?...It was the moment when I felt most present during the whole conversation."* (S28)

Reflection: This subcategory emerged during the second phase of the analysis process, the total relational presence collects different codes that characterize the experience of being fully present and attentive to the experience of the other person's experience and interaction

3.MAIN CATEGORY: INTERAFFECTIVE SPACE

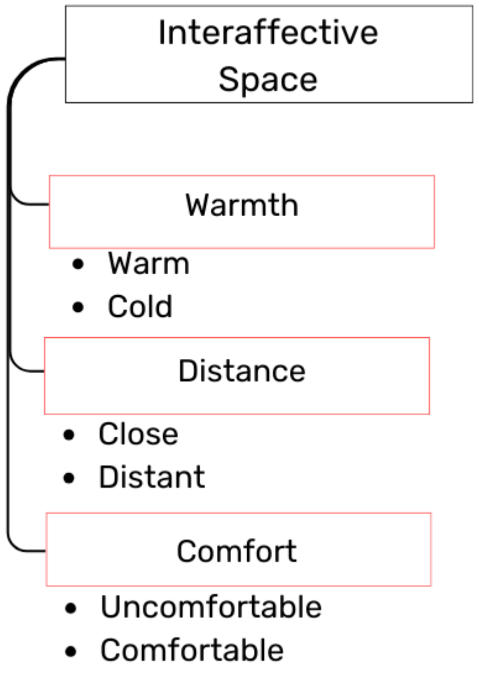

Description: It refers to the statements used to signify how participants perceived the emergent space within the actor-participant dynamic. It explores the qualitative aspects of the ambiance that unfolded during the interactions, encompassing both physical and emotional dimensions. During the interaction between the participants and the actor,

participants perceived spatial dynamics in various ways, ranging from proximity, comfort, and warmth to instances of distance, discomfort, and coolness.

Importance: It contributes to a holistic analysis of the context shared by interacting individuals. By considering both physical and emotional aspects, it provides valuable insights into participants' perceptions, feelings, and comfort levels during empathic interactions.

For example:

[Referring to interpersonal space during the interaction] *“I didn't feel that the atmosphere was tense. I feel like it was pretty fluid within how controlled the space was. Eh. But I feel it was very fluid. As I said, I think I, I felt like I could have even sat down and talked to him for a while... I felt very close to him...the space this space is comfortable for me.. I felt very close to him..”* (S17)

Reflection: This central category initially emerged with only two distinctive aspects: the perceived distance or closeness between interlocutors. After reviewing a larger number of experiences, its definition was enriched and expanded to include more subtle elements, such as sensations of warmth or coldness associated with the interpersonal space.

3.1 Grouped Subcategories: Warm-Comfortable-Close

Description: This subcategory refers to the perception of shared space within the participant-actor dynamic, where qualitative aspects of the ambiance, such as warmth, comfort, and closeness, are experienced.

Importance: It provides a framework to describe what it feels like to perceive an environment characterized by a sense of comfortable familiarity.

For example:

[Referring to interpersonal space during the interaction] *“Well, with the other questions," it seemed that little by little, we gained a bit more trust, more closeness, more warmth in itself. So here, the interaction was... warmer. It was easier and... it was not so complex." (S14)*

Reflection: This code emerged during the second phase of the analysis process. Initially, it comprised codes that referred to a space perceived as close, later merged with other attributes such as warmth and comfort.

3.2 Grouped Subcategories: Cold-Uncomfortable-Distant

Description: Refers to the participant's perception of the shared space in the participant-actor dynamic. In this context, qualitative aspects of the ambiance are characterized by a sense of coldness, discomfort, and distance. Participants describe experiences of spatial dynamics that convey a lack of warmth, a sense of discomfort, and a perceptible emotional distance within the interaction.

Importance: This subcategory gives qualities to the space shared between two people interacting and perceiving the interaction ambiance as uncomfortable. It provides space for phenomenological descriptions associated with qualities of coldness or distance, which are crucial for understanding experiential structures marked by greater disconnection.

For example:

[Referring to interpersonal space during the interaction] *"It looks like a cold, sober atmosphere. In the conversation, it was also just a question and answer, and no, there was nothing else. It was like well... how to confront something that generates suffering that is negative and how to feel it directly."* (S3)

[Referring to interpersonal space during interaction] "*When it all started, like... I felt it a little bit more like the atmosphere between the two of them was tense. And well, how distant."* (S19)

Reflection: Initially, it was formed by codes referring to a space perceived as distant, which later merged with other attributes such as uncomfortable or distant.

4. MAIN CATEGORY: DIS/ENGAGEMENT ACTS

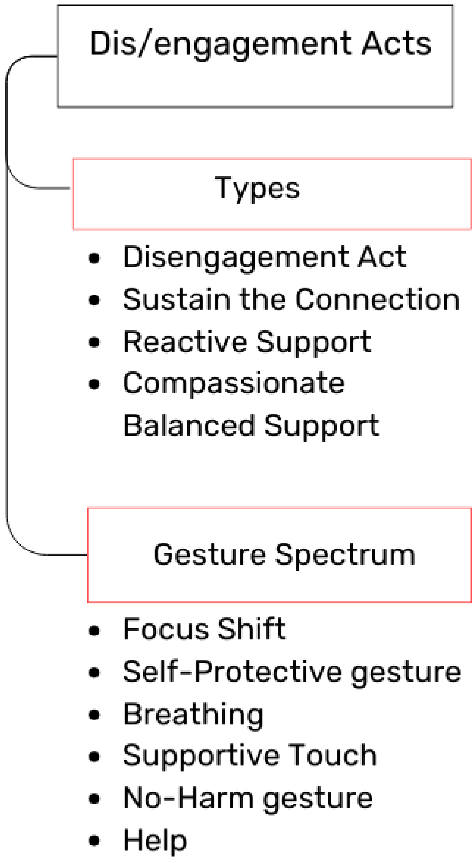

Description: Acts of dis/engagement are pre-reflective gestures performed by participants, intended to reinforce and nurture the actor-participant exchange or, conversely, to diminish or divert the interactive flow between the interlocutors. While some participants exhibit pre-reflective gestures such as focusing on maintaining the connection in the bond and providing support to the actor, others perform pre-reflective gestures intended to divert attention from the interaction.

Importance: This subcategory reveals nonreflexive strategies participants employ to manage emotional connection during empathic interactions. It provides detailed insights into how participants engage or disengage, contributing to a deeper understanding of empathic relational dynamics.

For example:

[Referring to the moment when the actor says he does not want to forget his wife] "*Well, I know that there is nothing in my hands to help him. Other than. Give him a smile. That when I smiled at them he smiled at me, uh... It was like wanting to give him my containment.*" (S15)

[Referring to the moment when the actor says he does not want to forget his wife] *“When that happens to me, I do something I always do with my fingers. I try to focus my attention on holding it together, but always with my hand. Because if I focus on my throat, like the knot I feel there, it gets worse. If I focus on the knot in my throat, it becomes harder to control. So, I focus on my fingers." (S23)*

Reflection: This category emerged from observations of various actions and gestures participants employed while interacting with the actor. It was developed during the second phase of the analysis process.

4.1 Subcategory: Disengagement Acts

Description: Pre-reflective gestures aimed at deliberately diverting attention from the interaction. This intentional disconnection from the development of the dynamics emerges at specific moments of suffering perceived by participants in the actor.

Importance: This subcategory is essential because it highlights intentional disengagement as a strategy used by participants during specific instances of perceived suffering.

For Example:
[Referring to the moment when the actor says he does not want to forget his wife] *"Whatever, maybe I kind of skipped it for a few seconds, like I stopped listening. I mean, I was listening, but without really analyzing what he was telling me... something to unlink his feelings from me."* (S27)

Reflection: This category emerged during the first phase of the analysis process, initially as codes referring to diverting the focus of attention as an intentional act.

4.2 Subcategory: Sustain the Connection

Description: Participants perform pre-reflective gestures to maintain their inner balance and interaction with others despite the distress they may feel. These gestures include intentional techniques such as focused breathing, attention to sensory perceptions (e.g., their hand), and deliberate control of emotional responses.

Importance: This subcategory provides concrete, pre-reflective strategies used by participants to maintain empathic connection during moments of distress. It captures subtle qualities that differentiate between the most disconnected and the most compassionate structures of experience.

For example:
[Referring to the final moments of the interaction] *"I mean, there comes a moment when something remains, but then you try to channel those sensations... I think that breathing a little bit is the only way to get rid of them."* (S7)

[Referring to the moment when the actor says he does not want to forget his wife] *"I paid more attention to what he was telling me. And I looked into his eyes... Sure, it fed my urge to cry, but I still concentrated on what he was saying."* (S9)

Reflection: This subcategory emerged during the second phase of the analysis process, identifying gestures of connection despite the participant's persistent distress.

4.3 Subcategory: Reactive support

Description: Distress-motivated support refers to the motivation to provide comfort to the actor, either through physical contact or supportive words, driven by distress. This motivation includes touch-based gestures, such as hugs or a reassuring hand on the shoulder, and a deeply ingrained motivation to help.

Importance: This subcategory differentiates the quality of support provided, which is often grouped as a unified dimension.

For example:
[Referring to the moment when the actor says he does not want to forget his wife] *"Then... I started to feel sadder because as he was talking, I could tell that he was also sad. At the same time, I noticed he was worried because of his body language. So that made me feel sorry for him. I even felt like hugging him, but obviously, I couldn't. I saw him as if he were my dad. I empathized with the situation and felt that he was already quite sad, affected by the word 'sad'."* (S13)
Reflection: This subcategory emerged during the second phase of the analysis process, after identifying experiences characterized by support gestures driven by anguish.

4.4 Subcategory: Compassionate Balanced support

Description: Corresponds to expressions where participants perform acts of compassionate support while effectively preserving their inner balance. In these cases, their motivation is directed toward comforting the actor through physical contact. Alternatively, some simply listen with a respectful attitude and the intention of alleviating the suffering of others through their presence. In both scenarios, the underlying motivations are rooted in emotional stability and self-control.

Importance: Compassionate Balanced Support is significant because it highlights the role of emotional balance in providing compassionate support. It enriches the understanding of diverse and adaptive empathic responses, illustrating how participants manage their emotions while offering comfort to the actor in real-time.

For example:
[Referring to the final moments of the interaction] *"Of course, I mean... because I don't have the confidence to tell him, he knows that this is not going to happen. Look, it's like having the intention to make him understand that it is a disease that can happen to anyone, and that family is family—it will always be there despite everything."* (S7)

Reflection: This subcategory emerged during the first phase of the analysis process, identifying gestures where participants balanced emotional regulation with actions aimed at supporting and helping the actor.

REFERENCES

Mihas, P., & Odum Institute. (2019). *Learn to build a codebook for a generic qualitative study*. SAGE Publications, Limit
