# Supplementary material for: From disconnection to compassion: a phenomenological exploration of embodied empathy in a face-to-face interaction
Source: Front Psychol. 2025 May 9;16:1522701. doi: 10.3389/fpsyg.2025.1522701 (PMC12098353; doi:10.3389/fpsyg.2025.1522701)
Supplement: Supplementary file 3 [file Data_Sheet_3.zip › Supplementary Material S3.html]

Supplementary Material 3: data analysis and results


Code 

- Show All Code
- Hide All Code

# Supplementary Material 3: data analysis and results

This HTML displays the results obtained from R after conducting coder
triangulation. Using R allowed summarizing the results in quantitative
terms (sample percentage). These results are based on the qualitative
analysis derived from the triangulation process, where each category and
its phase are linked to a phrase described by the participant. For
further details, please contact the authors of the article.

# 1 Results

## 1.1 Dynamic Lines

This section shows the quantification of the number of participants
who experience a specific category within each individual temporal
phase. In this way, the diachronic and synchronic elements of the
experience are combined.

|  |  |  |  |
| --- | --- | --- | --- |
| Percentage by First Order Category and Phase | | | |
| First Order | 1.1 Connection with the suffering | 1.2 Sensing the climax of anguish | 1.3 Navigating the Anguish |
| 2.1 Affective Quality | | | |
| --- | --- | --- | --- |
| 2.1.1 Relief | 2.380952 | 2.380952 | 7.142857 |
| 2.1.2 Anguish | 73.809524 | 95.238095 | 78.571429 |
| 2.1.3 Discomfort | 11.904762 | 9.523810 | 7.142857 |
| 2.1.4 Nervousness | 14.285714 | 9.523810 | 4.761905 |
| 2.2 Body feeling | | | |
| 2.2.1 Chest-Throat pressure | 47.619048 | 88.095238 | 61.904762 |
| 2.2.2 Watery eyes | 9.523810 | 23.809524 | 14.285714 |
| 2.2.3 Sadness feeling in the face | 2.380952 | 9.523810 | 4.761905 |
| 2.2.4 Open Heart feeling | 0.000000 | 4.761905 | 2.380952 |
| 2.2.5 a hollow feeling in the abdomen | 0.000000 | 7.142857 | 2.380952 |
| 2.3 General body tone | | | |
| 2.3.1 Tension | 14.285714 | 30.952381 | 14.285714 |
| 2.3.2 Heaviness | 0.000000 | 7.142857 | 2.380952 |
| 2.3.3 Agitation | 4.761905 | 7.142857 | 0.000000 |
| 2.3.4 Movement feeling | 7.142857 | 7.142857 | 4.761905 |
| 2.4 Intensity | | | |
| 2.4.1 High | 7.142857 | 92.857143 | 38.095238 |
| 2.4.2 Media | 38.095238 | 0.000000 | 33.333333 |
| 2.4.3 Low | 9.523810 | 4.761905 | 7.142857 |
| 2.4.4 Increasing | 47.619048 | 4.761905 | 0.000000 |
| 2.4.5 Decreasing Intensity | 0.000000 | 0.000000 | 35.714286 |
| 2.4.6 Intensity in Maintenance | 0.000000 | 2.380952 | 57.142857 |
| 3.0 Quality | | | |
| 3.0.1 Absence | 0.000000 | 2.380952 | 9.523810 |
| 3.0.2 Intermittent Presence | 19.047619 | 33.333333 | 38.095238 |
| 3.0.3 Full Presence | 28.571429 | 52.380952 | 45.238095 |
| 3.1 Attentional focus | | | |
| 3.1.2 Body language of the other | 35.714286 | 38.095238 | 30.952381 |
| 3.1.3 Unidirectional towards itself | 0.000000 | 2.380952 | 7.142857 |
| 3.1.4 Bidirectional | 23.809524 | 42.857143 | 28.571429 |
| 3.1.5 Unidirectional towards each other's context | 0.000000 | 4.761905 | 4.761905 |
| 3.2 Internal dialogue | | | |
| 3.2.1 Disconnected | 7.142857 | 11.904762 | 23.809524 |
| 3.2.2 Connected | 19.047619 | 59.523810 | 50.000000 |
| 3.3 Imagination | | | |
| 3.3.2 About the other | 4.761905 | 16.666667 | 9.523810 |
| 3.3.3 About himself | 9.523810 | 38.095238 | 21.428571 |
| 3.3.4 On the context | 2.380952 | 9.523810 | 9.523810 |
| 4.1 Types | | | |
| 4.1.1 Disengagement Act | 0.000000 | 11.904762 | 19.047619 |
| 4.1.2 Sustain the connection | 11.904762 | 35.714286 | 30.952381 |
| 4.1.3 Reactive Support | 0.000000 | 2.380952 | 21.428571 |
| 4.1.4 Compassionate balanced Support | 9.523810 | 7.142857 | 30.952381 |
| 4.2 Modality | | | |
| 4.2.1 Supportive Touch | 19.047619 | 19.047619 | 40.476190 |
| 4.2.2 Self-protective act | 2.380952 | 2.380952 | 2.380952 |
| 4.2.3 Help | 2.380952 | 14.285714 | 14.285714 |
| 4.2.4 Mantain the focus | 7.142857 | 11.904762 | 19.047619 |
| 4.2.5 Breathing | 2.380952 | 4.761905 | 9.523810 |
| 4.2.6 No harm | 7.142857 | 9.523810 | 26.190476 |
| 4.2.7 Focus Shift | 0.000000 | 11.904762 | 14.285714 |
| 5. Interpersonal Space | | | |
| 5.1 Comfortable | 28.571429 | 45.238095 | 35.714286 |
| 5.2 Close | 28.571429 | 38.095238 | 23.809524 |
| 5.3 Distant | 16.666667 | 23.809524 | 26.190476 |
| 5.4 Uncomfortable | 7.142857 | 9.523810 | 9.523810 |
| 5.5 Open | 0.000000 | 4.761905 | 4.761905 |
| 5.6 Warm | 16.666667 | 23.809524 | 19.047619 |

## 1.2 Four Structures of Experience Analysis

In the R analysis, the data analysis allows us to group the findings
into descriptions of specific elements, diachronic structures, and
dynamic lines. Here, we focus on a specific temporal phase and compare
all the elements across different structures. This comparison is
illustrated using a spider plot function in R, which displays the
percentage of each element according to the experiential structure in
Phase 3, as demonstrated in the analysis.

|  |  |  |  |  |  |  |  |  |  |  |  |  |  |  |  |  |  |  |  |  |  |  |  |  |  |  |  |  |  |  |  |  |  |  |  |  |  |  |  |  |  |  |  |  |  |  |
| --- | --- | --- | --- | --- | --- | --- | --- | --- | --- | --- | --- | --- | --- | --- | --- | --- | --- | --- | --- | --- | --- | --- | --- | --- | --- | --- | --- | --- | --- | --- | --- | --- | --- | --- | --- | --- | --- | --- | --- | --- | --- | --- | --- | --- | --- | --- |
| Percentage by Structure in Phase 3 | | | | | | | | | | | | | | | | | | | | | | | | | | | | | | | | | | | | | | | | | | | | | | |
|  | 2.1.1 Relief | 2.1.2 Anguish | 2.1.3 Discomfort | 2.1.4 Nervousness | 2.2.1 Chest-Throat pressure | 2.2.2 Watery eyes | 2.2.3 Sadness feeling in the face | 2.2.4 Open Heart feeling | 2.2.5 a hollow feeling in the abdomen | 2.3.1 Tension | 2.3.2 Heaviness | 2.3.4 Movement feeling | 2.4.1 High | 2.4.2 Media | 2.4.3 Low | 2.4.5 Decreasing Intensity | 2.4.6 Intensity in Maintenance | 3.0.1 Absence | 3.0.2 Intermittent Presence | 3.0.3 Full Presence | 3.1.2 Body language of the other | 3.1.3 Unidirectional towards itself | 3.1.4 Bidirectional | 3.1.5 Unidirectional towards each other's context | 3.2.1 Disconnected | 3.2.2 Connected | 3.3.2 About the other | 3.3.3 About himself | 3.3.4 On the context | 4.1.1 Disengagement Act | 4.1.2 Sustain the connection | 4.1.3 Reactive Support | 4.1.4 Compassionate balanced Support | 4.2.1 Supportive Touch | 4.2.2 Self-protective act | 4.2.3 Help | 4.2.4 Mantain the focus | 4.2.5 Breathing | 4.2.6 No harm | 4.2.7 Focus Shift | 5.1 Comfortable | 5.2 Close | 5.3 Distant | 5.4 Uncomfortable | 5.5 Open | 5.6 Warm |
| 0.1 Relational Disengagement | 33.33 | 66.67 | 50.00 | 16.67 | 33.33 | 16.67 | 0.00 | 0.00 | 0.00 | 16.67 | 0.00 | 0.00 | 33.33 | 16.67 | 33.33 | 50.00 | 50.00 | 66.67 | 33.33 | 0.00 | 0.00 | 50.00 | 33.33 | 16.67 | 66.67 | 16.67 | 0.00 | 33.33 | 33.33 | 83.33 | 0.00 | 0.00 | 0.00 | 0.00 | 0.00 | 0.00 | 0.00 | 0.00 | 0.00 | 66.67 | 16.67 | 0.00 | 83.33 | 16.67 | 0.00 | 0.00 |
| 0.4 Compassionate Support for suffering | 7.14 | 71.43 | 0.00 | 0.00 | 50.00 | 0.00 | 14.29 | 7.14 | 0.00 | 21.43 | 0.00 | 7.14 | 0.00 | 57.14 | 7.14 | 57.14 | 35.71 | 0.00 | 0.00 | 100.00 | 64.29 | 0.00 | 14.29 | 0.00 | 0.00 | 71.43 | 7.14 | 0.00 | 0.00 | 0.00 | 42.86 | 0.00 | 85.71 | 64.29 | 0.00 | 35.71 | 14.29 | 14.29 | 42.86 | 0.00 | 57.14 | 42.86 | 0.00 | 0.00 | 14.29 | 28.57 |
| 0.2 Persistent Angst | 0.00 | 92.31 | 0.00 | 0.00 | 76.92 | 30.77 | 0.00 | 0.00 | 7.69 | 15.38 | 0.00 | 0.00 | 61.54 | 30.77 | 0.00 | 30.77 | 61.54 | 0.00 | 61.54 | 30.77 | 23.08 | 0.00 | 38.46 | 7.69 | 30.77 | 53.85 | 15.38 | 30.77 | 7.69 | 15.38 | 30.77 | 0.00 | 7.69 | 7.69 | 7.69 | 0.00 | 23.08 | 15.38 | 7.69 | 15.38 | 30.77 | 15.38 | 23.08 | 7.69 | 0.00 | 15.38 |
| 0.3 Anguish Anchoring with Other-Oriented Support | 0.00 | 77.78 | 0.00 | 11.11 | 77.78 | 11.11 | 0.00 | 0.00 | 0.00 | 0.00 | 11.11 | 11.11 | 66.67 | 11.11 | 0.00 | 0.00 | 88.89 | 0.00 | 66.67 | 11.11 | 11.11 | 0.00 | 33.33 | 0.00 | 22.22 | 33.33 | 11.11 | 33.33 | 11.11 | 11.11 | 33.33 | 100.00 | 0.00 | 77.78 | 0.00 | 11.11 | 33.33 | 0.00 | 44.44 | 0.00 | 22.22 | 22.22 | 33.33 | 22.22 | 0.00 | 22.22 |

```
## png 
##   2
```

```
## png 
##   2
```

```
## png 
##   2
```

```
## png 
##   2
```

```
## # A tibble: 1 × 7
##   format width height colorspace matte filesize density
##   <chr>  <int>  <int> <chr>      <lgl>    <int> <chr>  
## 1 PNG     4200   4200 sRGB       FALSE        0 118x118
```

```
## null device 
##           1
```

## 1.3 Correlation between Phenomenological dimensions

The first step was to convert the main categories into an ordinal
scale that could be interpreted quantitatively as continuous. For
example, the main category ‘presence’ was interpreted on a scale from
lower to higher presence, with the subcategory ‘absence’ coded as 0,
‘intermittent presence’ as 1, and ‘full presence’ as 2. Similarly, this
approach was applied to engagement acts, with 0 representing
disengagement and 4 representing compassionate acts. This step required
careful interpretation, as not all main categories were suitable for
conversion into quantitative measures (e.g., affective quality). R
software made it easy to convert each subcategory into a numerical value
in the dataset for all subjects with case\_when function. This was
transferred to JASPT, and the data obtained from that application was
copied.

```
## # A tibble: 1 × 7
##   format width height colorspace matte filesize density
##   <chr>  <int>  <int> <chr>      <lgl>    <int> <chr>  
## 1 PNG     2439   1384 sRGB       TRUE    176689 57x57
```

```
## # A tibble: 1 × 7
##   format width height colorspace matte filesize density
##   <chr>  <int>  <int> <chr>      <lgl>    <int> <chr>  
## 1 PNG     1656   1250 sRGB       FALSE    93960 89x89
```

```
## # A tibble: 1 × 7
##   format width height colorspace matte filesize density
##   <chr>  <int>  <int> <chr>      <lgl>    <int> <chr>  
## 1 PNG     1656   1250 sRGB       FALSE    89127 89x89
```

# 2 Inter-rater Agreement

The inter-rater agreements by subject are presented. In the
inter-rater agreement, all annotations from the analysts that include
temporality and a phenomenological dimension are considered. The
percentage of agreement is calculated for each subject, and then all
agreements are averaged.

|  |  |
| --- | --- |
| Example of Computation of Agreement in R | |
| id | Agreement (%) |
| S1 | 76.00 |
| S10 | 76.47 |
| S11 | 78.79 |
| S12 | 73.91 |
| S13 | 92.11 |
| S14 | 68.29 |
| S15 | 82.86 |
| S16 | 71.15 |
| S17 | 83.78 |
| S18 | 94.44 |
| S19 | 70.45 |
| S2 | 64.52 |
| S20 | 79.41 |
| S21 | 93.33 |
| S22 | 78.57 |
| S23 | 94.44 |
| S24 | 92.50 |
| S25 | 90.62 |
| S26 | 90.32 |
| S27 | 81.82 |
| S28 | 91.18 |
| S29 | 88.89 |
| S3 | 81.25 |
| S30 | 60.00 |
| S31 | 68.00 |
| S32 | 75.61 |
| S33 | 78.38 |
| S34 | 85.19 |
| S35 | 95.83 |
| S36 | 90.48 |
| S37 | 65.71 |
| S38 | 79.07 |
| S39 | 61.11 |
| S4 | 80.00 |
| S40 | 100.00 |
| S41 | 78.57 |
| S42 | 80.00 |
| S5 | 85.19 |
| S6 | 90.32 |
| S7 | 70.97 |
| S8 | 87.88 |
| S9 | 88.10 |
| Average | 81.32 |
