# Supplementary material for: From disconnection to compassion: a phenomenological exploration of embodied empathy in a face-to-face interaction
Source: Front Psychol. 2025 May 9;16:1522701. doi: 10.3389/fpsyg.2025.1522701 (PMC12098353; doi:10.3389/fpsyg.2025.1522701)
Supplement: Supplementary file 3 [file Data_Sheet_3.zip › Supplementary Material S4.html]

Supplementary Material 4: Raw Data and Triangulation process


Code 

- Show All Code
- Hide All Code

# Supplementary Material 4: Raw Data and Triangulation process

This HML shows the process used to perform triangulation via R. All
of this data presents the raw evidence in Citations found in the
individual projects of Atlas Ti. Additionally, by filtering the values
with which there is agreement, it is possible to display all the data
used for the calculation of percentages and other procedures that are
detailed in the supplementary material. For the R code, please contact
the authors.

# 1 Subject-by-subject triangulation process

This shows the analysis of each study participant (S), indicating
where the codifications are in agreement or disagreement. These
codifications are always associated with a temporal phase of the
experience and were performed by two codifiers for each subject from a
pool of three codifiers. Each table allows filtering by category,
temporal phase, and agreement level for a faster process. When
disagreement was found (we filtered by disagreement in the last column),
we reviewed the codification in Atlas Ti within the interview and
categorized paragraph, and then discussed it.

## 1.1 S1

```
## Adding missing grouping variables: `id`
```

```
##  Percentage agreement (Tolerance=0)
## 
##  Subjects = 25 
##    Raters = 2 
##   %-agree = 76
```

```
##  Single Score Intraclass Correlation
## 
##    Model: oneway 
##    Type : agreement 
## 
##    Subjects = 25 
##      Raters = 2 
##      ICC(1) = -0.116
## 
##  F-Test, H0: r0 = 0 ; H1: r0 > 0 
##    F(24,25) = 0.792 , p = 0.715 
## 
##  95%-Confidence Interval for ICC Population Values:
##   -0.478 < ICC < 0.282
```

## 1.2 S2

```
##  Percentage agreement (Tolerance=0)
## 
##  Subjects = 31 
##    Raters = 2 
##   %-agree = 64.5
```

```
##  Single Score Intraclass Correlation
## 
##    Model: oneway 
##    Type : agreement 
## 
##    Subjects = 31 
##      Raters = 2 
##      ICC(1) = -0.2
## 
##  F-Test, H0: r0 = 0 ; H1: r0 > 0 
##    F(30,31) = 0.667 , p = 0.865 
## 
##  95%-Confidence Interval for ICC Population Values:
##   -0.51 < ICC < 0.159
```

## 1.3 S3

```
##  Percentage agreement (Tolerance=0)
## 
##  Subjects = 32 
##    Raters = 2 
##   %-agree = 81.2
```

```
##  Single Score Intraclass Correlation
## 
##    Model: oneway 
##    Type : agreement 
## 
##    Subjects = 32 
##      Raters = 2 
##      ICC(1) = -0.0877
## 
##  F-Test, H0: r0 = 0 ; H1: r0 > 0 
##    F(31,32) = 0.839 , p = 0.687 
## 
##  95%-Confidence Interval for ICC Population Values:
##   -0.416 < ICC < 0.262
```

## 1.4 S4

```
##  Percentage agreement (Tolerance=0)
## 
##  Subjects = 35 
##    Raters = 2 
##   %-agree = 80
```

```
##  Single Score Intraclass Correlation
## 
##    Model: oneway 
##    Type : agreement 
## 
##    Subjects = 35 
##      Raters = 2 
##      ICC(1) = -0.0968
## 
##  F-Test, H0: r0 = 0 ; H1: r0 > 0 
##    F(34,35) = 0.824 , p = 0.714 
## 
##  95%-Confidence Interval for ICC Population Values:
##   -0.41 < ICC < 0.238
```

## 1.5 S5

```
##  Percentage agreement (Tolerance=0)
## 
##  Subjects = 27 
##    Raters = 2 
##   %-agree = 85.2
```

```
##  Single Score Intraclass Correlation
## 
##    Model: oneway 
##    Type : agreement 
## 
##    Subjects = 27 
##      Raters = 2 
##      ICC(1) = -0.0612
## 
##  F-Test, H0: r0 = 0 ; H1: r0 > 0 
##    F(26,27) = 0.885 , p = 0.622 
## 
##  95%-Confidence Interval for ICC Population Values:
##   -0.421 < ICC < 0.318
```

## 1.6 S6

```
##  Percentage agreement (Tolerance=0)
## 
##  Subjects = 31 
##    Raters = 2 
##   %-agree = 90.3
```

```
##  Single Score Intraclass Correlation
## 
##    Model: oneway 
##    Type : agreement 
## 
##    Subjects = 31 
##      Raters = 2 
##      ICC(1) = -0.0345
## 
##  F-Test, H0: r0 = 0 ; H1: r0 > 0 
##    F(30,31) = 0.933 , p = 0.574 
## 
##  95%-Confidence Interval for ICC Population Values:
##   -0.376 < ICC < 0.317
```

## 1.7 S7

```
##  Percentage agreement (Tolerance=0)
## 
##  Subjects = 31 
##    Raters = 2 
##   %-agree = 71
```

```
##  Single Score Intraclass Correlation
## 
##    Model: oneway 
##    Type : agreement 
## 
##    Subjects = 31 
##      Raters = 2 
##      ICC(1) = -0.154
## 
##  F-Test, H0: r0 = 0 ; H1: r0 > 0 
##    F(30,31) = 0.733 , p = 0.801 
## 
##  95%-Confidence Interval for ICC Population Values:
##   -0.474 < ICC < 0.205
```

## 1.8 S8

```
##  Percentage agreement (Tolerance=0)
## 
##  Subjects = 33 
##    Raters = 2 
##   %-agree = 87.9
```

```
##  Single Score Intraclass Correlation
## 
##    Model: oneway 
##    Type : agreement 
## 
##    Subjects = 33 
##      Raters = 2 
##      ICC(1) = -0.0492
## 
##  F-Test, H0: r0 = 0 ; H1: r0 > 0 
##    F(32,33) = 0.906 , p = 0.609 
## 
##  95%-Confidence Interval for ICC Population Values:
##   -0.378 < ICC < 0.293
```

## 1.9 S9

```
##  Percentage agreement (Tolerance=0)
## 
##  Subjects = 42 
##    Raters = 2 
##   %-agree = 88.1
```

```
##  Single Score Intraclass Correlation
## 
##    Model: oneway 
##    Type : agreement 
## 
##    Subjects = 42 
##      Raters = 2 
##      ICC(1) = -0.0513
## 
##  F-Test, H0: r0 = 0 ; H1: r0 > 0 
##    F(41,42) = 0.902 , p = 0.628 
## 
##  95%-Confidence Interval for ICC Population Values:
##   -0.345 < ICC < 0.252
```

## 1.10 S10

```
##  Percentage agreement (Tolerance=0)
## 
##  Subjects = 34 
##    Raters = 2 
##   %-agree = 76.5
```

```
##  Single Score Intraclass Correlation
## 
##    Model: oneway 
##    Type : agreement 
## 
##    Subjects = 34 
##      Raters = 2 
##      ICC(1) = -0.119
## 
##  F-Test, H0: r0 = 0 ; H1: r0 > 0 
##    F(33,34) = 0.788 , p = 0.752 
## 
##  95%-Confidence Interval for ICC Population Values:
##   -0.432 < ICC < 0.222
```

## 1.11 S11

```
##  Percentage agreement (Tolerance=0)
## 
##  Subjects = 33 
##    Raters = 2 
##   %-agree = 78.8
```

```
##  Single Score Intraclass Correlation
## 
##    Model: oneway 
##    Type : agreement 
## 
##    Subjects = 33 
##      Raters = 2 
##      ICC(1) = -0.103
## 
##  F-Test, H0: r0 = 0 ; H1: r0 > 0 
##    F(32,33) = 0.812 , p = 0.721 
## 
##  95%-Confidence Interval for ICC Population Values:
##   -0.424 < ICC < 0.242
```

## 1.12 S12

```
##  Percentage agreement (Tolerance=0)
## 
##  Subjects = 23 
##    Raters = 2 
##   %-agree = 73.9
```

```
##  Single Score Intraclass Correlation
## 
##    Model: oneway 
##    Type : agreement 
## 
##    Subjects = 23 
##      Raters = 2 
##      ICC(1) = -0.128
## 
##  F-Test, H0: r0 = 0 ; H1: r0 > 0 
##    F(22,23) = 0.773 , p = 0.726 
## 
##  95%-Confidence Interval for ICC Population Values:
##   -0.501 < ICC < 0.289
```

## 1.13 S13

```
##  Percentage agreement (Tolerance=0)
## 
##  Subjects = 38 
##    Raters = 2 
##   %-agree = 92.1
```

```
##  Single Score Intraclass Correlation
## 
##    Model: oneway 
##    Type : agreement 
## 
##    Subjects = 38 
##      Raters = 2 
##      ICC(1) = -0.0278
## 
##  F-Test, H0: r0 = 0 ; H1: r0 > 0 
##    F(37,38) = 0.946 , p = 0.567 
## 
##  95%-Confidence Interval for ICC Population Values:
##   -0.338 < ICC < 0.289
```

## 1.14 S14

```
##  Percentage agreement (Tolerance=0)
## 
##  Subjects = 41 
##    Raters = 2 
##   %-agree = 68.3
```

```
##  Single Score Intraclass Correlation
## 
##    Model: oneway 
##    Type : agreement 
## 
##    Subjects = 41 
##      Raters = 2 
##      ICC(1) = -0.176
## 
##  F-Test, H0: r0 = 0 ; H1: r0 > 0 
##    F(40,41) = 0.7 , p = 0.869 
## 
##  95%-Confidence Interval for ICC Population Values:
##   -0.454 < ICC < 0.134
```

## 1.15 S15

```
##  Percentage agreement (Tolerance=0)
## 
##  Subjects = 35 
##    Raters = 2 
##   %-agree = 82.9
```

```
##  Single Score Intraclass Correlation
## 
##    Model: oneway 
##    Type : agreement 
## 
##    Subjects = 35 
##      Raters = 2 
##      ICC(1) = -0.0794
## 
##  F-Test, H0: r0 = 0 ; H1: r0 > 0 
##    F(34,35) = 0.853 , p = 0.678 
## 
##  95%-Confidence Interval for ICC Population Values:
##   -0.395 < ICC < 0.255
```

## 1.16 S16

```
##  Percentage agreement (Tolerance=0)
## 
##  Subjects = 52 
##    Raters = 2 
##   %-agree = 71.2
```

```
##  Single Score Intraclass Correlation
## 
##    Model: oneway 
##    Type : agreement 
## 
##    Subjects = 52 
##      Raters = 2 
##      ICC(1) = -0.159
## 
##  F-Test, H0: r0 = 0 ; H1: r0 > 0 
##    F(51,52) = 0.725 , p = 0.873 
## 
##  95%-Confidence Interval for ICC Population Values:
##   -0.411 < ICC < 0.116
```

## 1.17 S17

```
##  Percentage agreement (Tolerance=0)
## 
##  Subjects = 37 
##    Raters = 2 
##   %-agree = 83.8
```

```
##  Single Score Intraclass Correlation
## 
##    Model: oneway 
##    Type : agreement 
## 
##    Subjects = 37 
##      Raters = 2 
##      ICC(1) = -0.0746
## 
##  F-Test, H0: r0 = 0 ; H1: r0 > 0 
##    F(36,37) = 0.861 , p = 0.672 
## 
##  95%-Confidence Interval for ICC Population Values:
##   -0.383 < ICC < 0.25
```

## 1.18 S18

```
##  Percentage agreement (Tolerance=0)
## 
##  Subjects = 36 
##    Raters = 2 
##   %-agree = 94.4
```

```
##  Single Score Intraclass Correlation
## 
##    Model: oneway 
##    Type : agreement 
## 
##    Subjects = 36 
##      Raters = 2 
##      ICC(1) = -0.0145
## 
##  F-Test, H0: r0 = 0 ; H1: r0 > 0 
##    F(35,36) = 0.971 , p = 0.534 
## 
##  95%-Confidence Interval for ICC Population Values:
##   -0.335 < ICC < 0.31
```

## 1.19 S19

```
##  Percentage agreement (Tolerance=0)
## 
##  Subjects = 44 
##    Raters = 2 
##   %-agree = 70.5
```

```
##  Single Score Intraclass Correlation
## 
##    Model: oneway 
##    Type : agreement 
## 
##    Subjects = 44 
##      Raters = 2 
##      ICC(1) = -0.162
## 
##  F-Test, H0: r0 = 0 ; H1: r0 > 0 
##    F(43,44) = 0.721 , p = 0.857 
## 
##  95%-Confidence Interval for ICC Population Values:
##   -0.434 < ICC < 0.137
```

## 1.20 S20

```
##  Percentage agreement (Tolerance=0)
## 
##  Subjects = 34 
##    Raters = 2 
##   %-agree = 79.4
```

```
##  Single Score Intraclass Correlation
## 
##    Model: oneway 
##    Type : agreement 
## 
##    Subjects = 34 
##      Raters = 2 
##      ICC(1) = -0.1
## 
##  F-Test, H0: r0 = 0 ; H1: r0 > 0 
##    F(33,34) = 0.818 , p = 0.717 
## 
##  95%-Confidence Interval for ICC Population Values:
##   -0.417 < ICC < 0.24
```

## 1.21 S21

```
##  Percentage agreement (Tolerance=0)
## 
##  Subjects = 30 
##    Raters = 2 
##   %-agree = 93.3
```

```
##  Single Score Intraclass Correlation
## 
##    Model: oneway 
##    Type : agreement 
## 
##    Subjects = 30 
##      Raters = 2 
##      ICC(1) = 0.798
## 
##  F-Test, H0: r0 = 0 ; H1: r0 > 0 
##    F(29,30) = 8.9 , p = 2.5e-08 
## 
##  95%-Confidence Interval for ICC Population Values:
##   0.621 < ICC < 0.898
```

## 1.22 S22

```
##  Percentage agreement (Tolerance=0)
## 
##  Subjects = 42 
##    Raters = 2 
##   %-agree = 78.6
```

```
##  Single Score Intraclass Correlation
## 
##    Model: oneway 
##    Type : agreement 
## 
##    Subjects = 42 
##      Raters = 2 
##      ICC(1) = -0.108
## 
##  F-Test, H0: r0 = 0 ; H1: r0 > 0 
##    F(41,42) = 0.805 , p = 0.756 
## 
##  95%-Confidence Interval for ICC Population Values:
##   -0.394 < ICC < 0.198
```

## 1.23 S23

```
##  Percentage agreement (Tolerance=0)
## 
##  Subjects = 36 
##    Raters = 2 
##   %-agree = 94.4
```

```
##  Single Score Intraclass Correlation
## 
##    Model: oneway 
##    Type : agreement 
## 
##    Subjects = 36 
##      Raters = 2 
##      ICC(1) = -0.0145
## 
##  F-Test, H0: r0 = 0 ; H1: r0 > 0 
##    F(35,36) = 0.971 , p = 0.534 
## 
##  95%-Confidence Interval for ICC Population Values:
##   -0.335 < ICC < 0.31
```

## 1.24 S24

```
##  Percentage agreement (Tolerance=0)
## 
##  Subjects = 40 
##    Raters = 2 
##   %-agree = 92.5
```

```
##  Single Score Intraclass Correlation
## 
##    Model: oneway 
##    Type : agreement 
## 
##    Subjects = 40 
##      Raters = 2 
##      ICC(1) = 0.829
## 
##  F-Test, H0: r0 = 0 ; H1: r0 > 0 
##    F(39,40) = 10.7 , p = 6.39e-12 
## 
##  95%-Confidence Interval for ICC Population Values:
##   0.702 < ICC < 0.906
```

## 1.25 S25

```
##  Percentage agreement (Tolerance=0)
## 
##  Subjects = 32 
##    Raters = 2 
##   %-agree = 90.6
```

```
##  Single Score Intraclass Correlation
## 
##    Model: oneway 
##    Type : agreement 
## 
##    Subjects = 32 
##      Raters = 2 
##      ICC(1) = -0.0333
## 
##  F-Test, H0: r0 = 0 ; H1: r0 > 0 
##    F(31,32) = 0.935 , p = 0.573 
## 
##  95%-Confidence Interval for ICC Population Values:
##   -0.37 < ICC < 0.313
```

## 1.26 S26

```
##  Percentage agreement (Tolerance=0)
## 
##  Subjects = 31 
##    Raters = 2 
##   %-agree = 90.3
```

```
##  Single Score Intraclass Correlation
## 
##    Model: oneway 
##    Type : agreement 
## 
##    Subjects = 31 
##      Raters = 2 
##      ICC(1) = -0.0345
## 
##  F-Test, H0: r0 = 0 ; H1: r0 > 0 
##    F(30,31) = 0.933 , p = 0.574 
## 
##  95%-Confidence Interval for ICC Population Values:
##   -0.376 < ICC < 0.317
```

## 1.27 S27

```
##  Percentage agreement (Tolerance=0)
## 
##  Subjects = 44 
##    Raters = 2 
##   %-agree = 81.8
```

```
##  Single Score Intraclass Correlation
## 
##    Model: oneway 
##    Type : agreement 
## 
##    Subjects = 44 
##      Raters = 2 
##      ICC(1) = 0.64
## 
##  F-Test, H0: r0 = 0 ; H1: r0 > 0 
##    F(43,44) = 4.56 , p = 8.93e-07 
## 
##  95%-Confidence Interval for ICC Population Values:
##   0.428 < ICC < 0.786
```

## 1.28 S28

```
##  Percentage agreement (Tolerance=0)
## 
##  Subjects = 34 
##    Raters = 2 
##   %-agree = 91.2
```

```
##  Single Score Intraclass Correlation
## 
##    Model: oneway 
##    Type : agreement 
## 
##    Subjects = 34 
##      Raters = 2 
##      ICC(1) = -0.0312
## 
##  F-Test, H0: r0 = 0 ; H1: r0 > 0 
##    F(33,34) = 0.939 , p = 0.571 
## 
##  95%-Confidence Interval for ICC Population Values:
##   -0.358 < ICC < 0.304
```

## 1.29 S29

```
##  Percentage agreement (Tolerance=0)
## 
##  Subjects = 36 
##    Raters = 2 
##   %-agree = 88.9
```

```
##  Single Score Intraclass Correlation
## 
##    Model: oneway 
##    Type : agreement 
## 
##    Subjects = 36 
##      Raters = 2 
##      ICC(1) = -0.0448
## 
##  F-Test, H0: r0 = 0 ; H1: r0 > 0 
##    F(35,36) = 0.914 , p = 0.604 
## 
##  95%-Confidence Interval for ICC Population Values:
##   -0.361 < ICC < 0.282
```

## 1.30 S30

```
##  Percentage agreement (Tolerance=0)
## 
##  Subjects = 35 
##    Raters = 2 
##   %-agree = 60
```

```
##  Single Score Intraclass Correlation
## 
##    Model: oneway 
##    Type : agreement 
## 
##    Subjects = 35 
##      Raters = 2 
##      ICC(1) = -0.236
## 
##  F-Test, H0: r0 = 0 ; H1: r0 > 0 
##    F(34,35) = 0.618 , p = 0.918 
## 
##  95%-Confidence Interval for ICC Population Values:
##   -0.522 < ICC < 0.099
```

## 1.31 S31

```
##  Percentage agreement (Tolerance=0)
## 
##  Subjects = 25 
##    Raters = 2 
##   %-agree = 68
```

```
##  Single Score Intraclass Correlation
## 
##    Model: oneway 
##    Type : agreement 
## 
##    Subjects = 25 
##      Raters = 2 
##      ICC(1) = -0.171
## 
##  F-Test, H0: r0 = 0 ; H1: r0 > 0 
##    F(24,25) = 0.708 , p = 0.799 
## 
##  95%-Confidence Interval for ICC Population Values:
##   -0.52 < ICC < 0.23
```

## 1.32 S32

```
##  Percentage agreement (Tolerance=0)
## 
##  Subjects = 41 
##    Raters = 2 
##   %-agree = 75.6
```

```
##  Single Score Intraclass Correlation
## 
##    Model: oneway 
##    Type : agreement 
## 
##    Subjects = 41 
##      Raters = 2 
##      ICC(1) = -0.127
## 
##  F-Test, H0: r0 = 0 ; H1: r0 > 0 
##    F(40,41) = 0.775 , p = 0.789 
## 
##  95%-Confidence Interval for ICC Population Values:
##   -0.413 < ICC < 0.183
```

## 1.33 S33

```
##  Percentage agreement (Tolerance=0)
## 
##  Subjects = 37 
##    Raters = 2 
##   %-agree = 78.4
```

```
##  Single Score Intraclass Correlation
## 
##    Model: oneway 
##    Type : agreement 
## 
##    Subjects = 37 
##      Raters = 2 
##      ICC(1) = -0.108
## 
##  F-Test, H0: r0 = 0 ; H1: r0 > 0 
##    F(36,37) = 0.806 , p = 0.741 
## 
##  95%-Confidence Interval for ICC Population Values:
##   -0.411 < ICC < 0.219
```

## 1.34 S34

```
##  Percentage agreement (Tolerance=0)
## 
##  Subjects = 27 
##    Raters = 2 
##   %-agree = 85.2
```

```
##  Single Score Intraclass Correlation
## 
##    Model: oneway 
##    Type : agreement 
## 
##    Subjects = 27 
##      Raters = 2 
##      ICC(1) = -0.0612
## 
##  F-Test, H0: r0 = 0 ; H1: r0 > 0 
##    F(26,27) = 0.885 , p = 0.622 
## 
##  95%-Confidence Interval for ICC Population Values:
##   -0.421 < ICC < 0.318
```

## 1.35 S35

```
##  Percentage agreement (Tolerance=0)
## 
##  Subjects = 24 
##    Raters = 2 
##   %-agree = 95.8
```

```
##  Single Score Intraclass Correlation
## 
##    Model: oneway 
##    Type : agreement 
## 
##    Subjects = 24 
##      Raters = 2 
##      ICC(1) = 0
## 
##  F-Test, H0: r0 = 0 ; H1: r0 > 0 
##    F(23,24) = 1 , p = 0.499 
## 
##  95%-Confidence Interval for ICC Population Values:
##   -0.391 < ICC < 0.394
```

## 1.36 S36

```
##  Percentage agreement (Tolerance=0)
## 
##  Subjects = 21 
##    Raters = 2 
##   %-agree = 90.5
```

```
##  Single Score Intraclass Correlation
## 
##    Model: oneway 
##    Type : agreement 
## 
##    Subjects = 21 
##      Raters = 2 
##      ICC(1) = -0.0256
## 
##  F-Test, H0: r0 = 0 ; H1: r0 > 0 
##    F(20,21) = 0.95 , p = 0.544 
## 
##  95%-Confidence Interval for ICC Population Values:
##   -0.437 < ICC < 0.399
```

## 1.37 S37

```
##  Percentage agreement (Tolerance=0)
## 
##  Subjects = 35 
##    Raters = 2 
##   %-agree = 65.7
```

```
##  Single Score Intraclass Correlation
## 
##    Model: oneway 
##    Type : agreement 
## 
##    Subjects = 35 
##      Raters = 2 
##      ICC(1) = -0.193
## 
##  F-Test, H0: r0 = 0 ; H1: r0 > 0 
##    F(34,35) = 0.676 , p = 0.871 
## 
##  95%-Confidence Interval for ICC Population Values:
##   -0.488 < ICC < 0.144
```

## 1.38 S38

```
##  Percentage agreement (Tolerance=0)
## 
##  Subjects = 43 
##    Raters = 2 
##   %-agree = 79.1
```

```
##  Single Score Intraclass Correlation
## 
##    Model: oneway 
##    Type : agreement 
## 
##    Subjects = 43 
##      Raters = 2 
##      ICC(1) = -0.105
## 
##  F-Test, H0: r0 = 0 ; H1: r0 > 0 
##    F(42,43) = 0.81 , p = 0.753 
## 
##  95%-Confidence Interval for ICC Population Values:
##   -0.388 < ICC < 0.197
```

## 1.39 S39

```
##  Percentage agreement (Tolerance=0)
## 
##  Subjects = 36 
##    Raters = 2 
##   %-agree = 61.1
```

```
##  Single Score Intraclass Correlation
## 
##    Model: oneway 
##    Type : agreement 
## 
##    Subjects = 36 
##      Raters = 2 
##      ICC(1) = -0.228
## 
##  F-Test, H0: r0 = 0 ; H1: r0 > 0 
##    F(35,36) = 0.629 , p = 0.914 
## 
##  95%-Confidence Interval for ICC Population Values:
##   -0.512 < ICC < 0.103
```

## 1.40 S40

```
##  Percentage agreement (Tolerance=0)
## 
##  Subjects = 28 
##    Raters = 2 
##   %-agree = 100
```

```
##  Single Score Intraclass Correlation
## 
##    Model: oneway 
##    Type : agreement 
## 
##    Subjects = 28 
##      Raters = 2 
##      ICC(1) = NaN
## 
##  F-Test, H0: r0 = 0 ; H1: r0 > 0 
##    F(27,28) = NaN , p = NaN 
## 
##  95%-Confidence Interval for ICC Population Values:
##   NaN < ICC < NaN
```

## 1.41 S42

```
##  Percentage agreement (Tolerance=0)
## 
##  Subjects = 28 
##    Raters = 2 
##   %-agree = 78.6
```

```
##  Single Score Intraclass Correlation
## 
##    Model: oneway 
##    Type : agreement 
## 
##    Subjects = 28 
##      Raters = 2 
##      ICC(1) = -0.102
## 
##  F-Test, H0: r0 = 0 ; H1: r0 > 0 
##    F(27,28) = 0.815 , p = 0.702 
## 
##  95%-Confidence Interval for ICC Population Values:
##   -0.448 < ICC < 0.273
```

## 1.42 S43

```
##  Percentage agreement (Tolerance=0)
## 
##  Subjects = 40 
##    Raters = 2 
##   %-agree = 80
```

```
##  Single Score Intraclass Correlation
## 
##    Model: oneway 
##    Type : agreement 
## 
##    Subjects = 40 
##      Raters = 2 
##      ICC(1) = -0.0986
## 
##  F-Test, H0: r0 = 0 ; H1: r0 > 0 
##    F(39,40) = 0.821 , p = 0.731 
## 
##  95%-Confidence Interval for ICC Population Values:
##   -0.392 < ICC < 0.215
```
